# Evaluation of the quality and quantity of artificial intelligence-generated responses about anesthesia and surgery: using ChatGPT 3.5 and 4.0

**DOI:** 10.3389/fmed.2024.1400153

**Published:** 2024-07-11

**Authors:** Jisun Choi, Ah Ran Oh, Jungchan Park, Ryung A. Kang, Seung Yeon Yoo, Dong Jae Lee, Kwangmo Yang

**Affiliations:** ^1^Department of Anesthesiology and Pain Medicine, Samsung Medical Center, Sungkyunkwan University School of Medicine, Seoul, Republic of Korea; ^2^Center for Health Promotion, Samsung Medical Center, Sungkyunkwan University School of Medicine, Seoul, Republic of Korea

**Keywords:** ChatGPT, artificial intelligence, quality, quantity, AI chatbot

## Abstract

**Introduction:**

The large-scale artificial intelligence (AI) language model chatbot, Chat Generative Pre-Trained Transformer (ChatGPT), is renowned for its ability to provide data quickly and efficiently. This study aimed to assess the medical responses of ChatGPT regarding anesthetic procedures.

**Methods:**

Two anesthesiologist authors selected 30 questions representing inquiries patients might have about surgery and anesthesia. These questions were inputted into two versions of ChatGPT in English. A total of 31 anesthesiologists then evaluated each response for quality, quantity, and overall assessment, using 5-point Likert scales. Descriptive statistics summarized the scores, and a paired sample *t*-test compared ChatGPT 3.5 and 4.0.

**Results:**

Regarding quality, “appropriate” was the most common rating for both ChatGPT 3.5 and 4.0 (40 and 48%, respectively). For quantity, responses were deemed “insufficient” in 59% of cases for 3.5, and “adequate” in 69% for 4.0. In overall assessment, 3 points were most common for 3.5 (36%), while 4 points were predominant for 4.0 (42%). Mean quality scores were 3.40 and 3.73, and mean quantity scores were − 0.31 (between insufficient and adequate) and 0.03 (between adequate and excessive), respectively. The mean overall score was 3.21 for 3.5 and 3.67 for 4.0. Responses from 4.0 showed statistically significant improvement in three areas.

**Conclusion:**

ChatGPT generated responses mostly ranging from appropriate to slightly insufficient, providing an overall average amount of information. Version 4.0 outperformed 3.5, and further research is warranted to investigate the potential utility of AI chatbots in assisting patients with medical information.

## 1 Introduction

Each year, approximately 4.2 million patients worldwide undergo surgical procedures under anesthesia, with reported mortality rates of 2.75% within 30 days after various surgical operations, and anesthesia-related deaths occurring at a rate of 1.72 per 10,000 procedures (1, 2). Patients facing surgery often experience anxiety and seek explanations from healthcare professionals (3). However, these explanations may be perceived as insufficient, leading patients to turn to online sources for additional information. Regrettably, online information is not always reliable, and when patients encounter incorrect or misleading data, it can potentially escalate anxiety and negatively impact surgical outcomes (4).

In November 2022, a groundbreaking artificial intelligence (AI) language model chatbot named ChatGPT was released. Unlike conventional chatbots, it is known to analyze, comprehend, and learn from text to generate human-like answers, allowing direct and meaningful interactions with users and facilitating the exchange of information (5). Impressively, ChatGPT passed the United States Medical Licensing Examination and holds the potential to offer high-level and prompt responses concerning medical information (6, 7). Ongoing efforts by researchers, educators, and professionals aim to implement ChatGPT in diverse domains, spanning from composing medical papers to educational settings (8, 9). There is a strong anticipation that ChatGPT could contribute significantly, either as a supplementary tool or, potentially, as a partial replacement for the roles of medical experts. However, few studies have specifically assessed the accuracy and relevance of medical information provided by chatbots for the general population. Furthermore, the absence of evaluations on the information provided by ChatGPT regarding anesthetic procedures underscores the necessity for well-designed investigations to assess its effectiveness in this specific medical field. Therefore, our study aims to assess the appropriateness of medical information generated by ChatGPT and determine whether this AI chatbot can effectively offer rapid and accessible medical advice to patients preparing for surgery and anesthesia. Additionally, we will compare the responses of ChatGPT 3.5 and the latest model, 4.0, to discern which version proves more beneficial.

## 2 Materials and methods

This study was initially submitted to the institutional review board (IRB) at our institution, seeking ethical review. After careful consideration, the IRB determined that formal review was not required, as the study does not involve human subjects. The focus lies on the analysis of data generated by the “ChatGPT” program, which does not necessitate direct interaction or involvement with individuals. The research strictly adheres to all applicable ethical guidelines and regulations, ensuring the confidentiality and privacy of any data used during the analysis process.

### 2.1 Study design

To address common inquiries of individuals anticipating surgery or anesthesia, two anesthesiologists crafted a set of 30 questions. These questions encompassed various topics, including the type of anesthesia, preoperative preparation, preanesthetic evaluation criteria, and the surgical and anesthetic recovery process. The formulation of these questions involved referencing educational materials provided by the Korean Society of Anesthesiologists for the public, in addition to information from relevant textbooks and research papers concerning preoperative assessments in anesthesia. All questions were composed in English and entered into both the freely accessible ChatGPT 3.5 version and the paid-only 4.0 version. Each interaction was labeled as a “new chat.”

**Figure 1:**
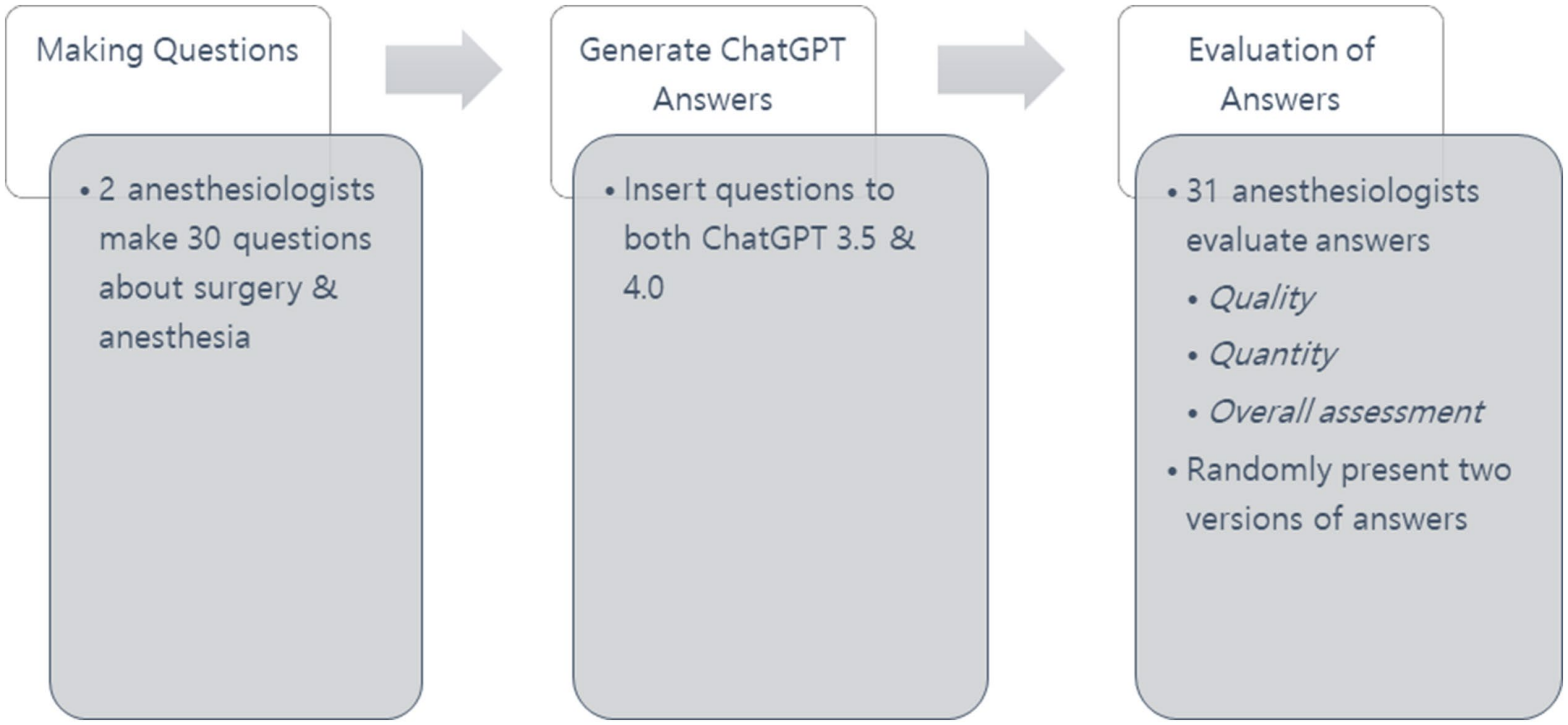
Research process.

Response evaluations were carried out by 31 anesthesiologists from university hospitals in Korea. To maintain impartiality, the two authors who created the questions did not participate in the evaluation process. Each evaluator received two versions of the answer for each question without noticing which ChatGPT version produced it. The presentation of responses was randomized to ensure unbiased evaluation. The overall study design and flow are presented in Figure 1.

### 2.2 Evaluation: quality, quantity, overall assessment

Responses were evaluated based on three criteria: quality, quantity, and overall score. For quality evaluation, the appropriateness of responses was assessed using a 5-point Likert scale (1—very inappropriate, 2—inappropriate, 3—average, 4—appropriate, 5—very appropriate). Quantity evaluation determined if responses were insufficient or excessive, utilizing a 5-point Likert scale (−2—very insufficient, −1—insufficient, 0—adequate, 1—excessive, 2—very excessive). The overall assessment represented a comprehensive evaluation of the responses, where participants rated how well the information was provided on a 5-point Likert scale, ranging from 1 (the response should not be provided) to 5 (the response is perfect).

To evaluate in which areas ChatGPT provides better responses, the questions were divided into four categories, and additional analysis was conducted: (1) General questions about anesthesia, (2) Preoperative preparation, (3) Pre-anesthetic evaluation, (4) Postoperative recovery process.

### 2.3 Statistical analysis

Mean values with standard deviations were calculated for each score based on the responses from the 31 participants. A paired *t*-test was then conducted to compare the values between the 3.5 and 4.0 versions. A significance level of *p* < 0.05 was employed to determine if there was a statistically significant difference. All statistical analyses were carried out using SPSS statistical software (IBM SPSS Statistics 20; Chicago, IL, United States).

## 3 Results

The entire set of questions and the results of the response evaluation by 31 experienced anesthesiologists are presented in Table 1. The full text of the responses generated by ChatGPT 3.5 and 4.0 is provided in Supplementary Table S1. The overall distribution of response evaluations according to the version of ChatGPT is summarized in Table 2.

**Table 1:** Questions and the results of response evaluations by experts.

No.	Question	ChatGPT 3.5			ChatGPT 4.0		
		Quality	Quantity	overall	Quality	Quantity	overall
1	What is general anesthesia and how does it work?	3.61	−0.1	3.58	3.84	0.19	3.68
2	What are the possible complications after general anesthesia?	3.39	−0.06	3.32	4.29	0.23	4.23
3	What is the mortality rate from anesthesia in healthy people?	3.32	−0.19	3.32	4.1	0.06	4.03
4	What is “awakening under anesthesia”?	3.65	−0.19	3.58	3.68	0.03	3.77
5	What are the side effects of spinal anesthesia?	3.52	−0.32	3.52	4.29	0.39	4.1
6	How long does it take for sensation to return after spinal anesthesia?	3.32	−0.13	3.1	3.84	−0.03	3.94
7	When is epidural anesthesia performed?	3.42	−0.26	3.19	4.06	0.16	3.97
8	What are some of the underlying conditions that can be dangerous when receiving anesthesia?	3.71	−0.29	3.42	3.94	0.16	3.97
9	What items should be evaluated before surgery in a patient with reduced mobility?	3.29	−0.39	2.87	4.03	0.13	4.06
10	What items should be evaluated before surgery in patients with alcohol dependence?	3.45	−0.16	3.39	4	0.42	3.81
11	Is it safe for pregnant women to receive general anesthesia?	3.39	−0.52	3.16	3.97	0.26	3.87
12	Why do I need to fast before surgery?	3.13	−0.19	2.94	3.71	0.06	3.74
13	How long do I need to fast before surgery?	3.42	−0.29	3.32	3.77	0	3.87
14	Do I need to quit smoking before anesthesia?	3.29	−0.55	3.03	4.03	0.13	4.06
15	How long do I need to quit smoking to be safe during surgery?	3.35	−0.19	3.39	3.61	−0.16	3.61
16	The day before surgery, I had a fever, cough, and phlegm. Can I have general anesthesia?	3.58	−0.06	3.74	2.87	−0.87	2.68
17	I am taking anticoagulants. How long should I stop before surgery?	3	−0.71	2.71	3.42	−0.16	3.42
18	What medications do I need to keep taking before surgery until the day of surgery?	3.23	−0.58	2.9	3.52	−0.23	3.52
19	What diseases or conditions require cardiac evaluation before surgery?	3.42	−0.45	3.16	3.74	0.26	3.68
20	What diseases or conditions require lung-related evaluation before surgery?	3.52	−0.32	3.19	3.71	0.16	3.71
21	What kind of evaluation is needed for hypertensive patients before surgery?	3.39	−0.26	3.16	3.68	0.13	3.74
22	A patient with a pacemaker is scheduled to receive general anesthesia. What should I watch out for?	3.52	−0.35	3.16	3.77	0.23	3.58
23	What kind of evaluation do asthma patients need before surgery?	3.61	−0.26	3.29	3.84	0.23	3.87
24	What precautions should be taken before anesthesia for people with poor blood sugar control?	3.58	−0.32	3.29	3.81	0.29	3.87
25	What kind of evaluation do patients with psychiatric problems such as anxiety and depression need before surgery?	3.45	−0.23	3.26	3.55	0.26	3.55
26	What is the most commonly used drug for sedation or anesthesia?	3.39	−0.48	3.06	3.03	−0.71	2.61
27	How can I manage pain after surgery?	2.81	−0.65	2.58	3.71	0	3.45
28	How can I control nausea and vomiting after surgery?	3.03	−0.48	2.84	3.23	−0.23	3.03
29	How many hours after going up from the recovery room to the ward can I eat?	3.45	−0.23	3.26	3.16	−0.45	2.87
30	Sore throat after general anesthesia. What should I do?	3.61	0	3.52	3.77	0	3.77

Quality (5-point Likert scale; 1 very inappropriate, 2 inappropriate, 3 average, 4 appropriate, 5 very appropriate). Quantity (5 Likert; −2 very insufficient, −1 insufficient, 0 adequate, 1 excessive, 2 very excessive). Overall assessment (5 Likert; 1 should not be an answer to 5 perfect answer).

**Table 2:** Distribution of response evaluation by experts according to version of ChatGPT.

		ChatGPT 3.5 (%)	ChatGPT 4.0 (%)
Quality	1: Very inappropriate	1	1
	2: Inappropriate	13	6
	3: Average	38	29
	4: Appropriate	40	48
	5: Very appropriate	7	16
Quantity	−2: Very insufficient	2	1
	−1: insufficient	33	13
	0: Adequate	59	69
	1: Excessive	6	17
	2: Very excessive	0	1
Overall assessment	1: Worst	2	1
	2	22	9
	3	36	29
	4	31	42
	5: Best	9	18

### 3.1 ChatGPT 3.5

The mean score for the “quality” of the 30 answers generated by ChatGPT 3.5 was 3.40 (±0.20), indicating a level between “average” and “appropriate.” The highest percentage, 40%, was observed for the score of 4, indicating “appropriate,” while the lowest percentage, 1%, was recorded for the score of 1, indicating “very inappropriate.” Regarding the “quantity” of the answers, the score of 0, indicating “adequate,” had the highest percentage at 59%, and the mean score was −0.31 (±0.18), indicating a value between “insufficient” and “adequate.” The mean score for the “overall assessment” was 3.21 (±0.27), with the highest percentage (36%) observed for the score of 3.

### 3.2 ChatGPT 4.0

The mean score for the “quality” of the answers generated by ChatGPT 4.0 was 3.73 (±0.34), similar to ChatGPT 3.5, indicating a level between “average” and “appropriate.” The score of 4 had the highest percentage, with 48% of respondents selecting it. Regarding the “quantity” of the answers, the mean score was 0.03 (±0.30), suggesting an evaluation between “adequate” and “excessive.” Notably, a significant proportion of 69% rated the responses as “adequate,” scoring 0 on the scale. For the “overall assessment,” the mean score was 3.67 (±0.40), with the highest percentage of 42% of participants giving a score of 4, reflecting positive feedback on the responses generated by ChatGPT 4.0.

### 3.3 Evaluation by category

When examined by category, ChatGPT’s performance for “General questions about anesthesia” showed that in version 3.5, 52% of responses were of appropriate or higher quality, 57% were of adequate quantity, and 10% received the best grade. In version 4.0, these figures were 76, 67, and 23%, respectively. For “Preoperative preparation,” ChatGPT 3.5 achieved 45% in quality, 60% in quantity, and 10% for the best grade, while version 4.0 achieved 57, 66, and 18%. Regarding “Pre-anesthetic evaluation,” ChatGPT 3.5 scored 49% in quality, 60% in quantity, and 6% for the best grade, whereas version 4.0 scored 67, 72, and 18%. For the “Postoperative recovery process,” ChatGPT 3.5 achieved 40% in quality, 60% in quantity, and 8% for the best grade, while version 4.0 achieved 52, 70, and 13% (Table 3). In all categories, version 4.0 showed higher percentages. However, except for responses on “Preoperative evaluation” in ChatGPT 4.0, there were instances where responses were rated as very inappropriate, very insufficient, or the worst in all categories.

**Table 3:** Evaluation of responses related to anesthesia and surgery generated by two versions of ChatGPT by question type.

		ChatGPT 3.5 (%)	ChatGPT 4.0 (%)
General questions about anesthesia (*N* = 8)			
Quality	1: Very inappropriate	2	1
	2: Inappropriate	13	4
	3: Average	33	19
	4: Appropriate	43	54
	5: Very appropriate	9	22
Quantity	−2: Very insufficient	2	1
	−1: Insufficient	33	12
	0: Adequate	57	67
	1: Excessive	8	19
	2: Very excessive	0	1
Overall assessment	1: Worst	1	2
	2	19	8
	3	34	23
	4	35	43
	5: Best	10	23
Preoperative preparation (*N* = 8)			
Quality	1: Very inappropriate	2	1
	2: Inappropriate	17	9
	3: Average	36	33
	4: Appropriate	38	44
	5: Very appropriate	7	13
Quantity	−2: Very insufficient	2	2
	−1: Insufficient	34	21
	0: Adequate	60	66
	1: Excessive	3	11
	2: Very excessive	0	0
Overall assessment	1: Worst	4	1
	2	25	13
	3	33	31
	4	29	38
	5: Best	10	18
Pre-anesthetic evaluation (*N* = 9)			
Quality	1: Very inappropriate	0	0
	2: Inappropriate	7	2
	3: Average	44	30
	4: Appropriate	42	52
	5: Very appropriate	7	15
Quantity	−2: Very insufficient	1	0
	−1: Insufficient	34	3
	0: Adequate	60	72
	1: Excessive	5	24
	2: Very excessive	0	1
Overall assessment	1: Worst	1	0
	2	18	5
	3	44	31
	4	29	47
	5: Best	6	18
Postoperative recovery process (*N* = 5)			
Quality	1: Very inappropriate	1	1
	2: Inappropriate	20	10
	3: Average	39	37
	4: Appropriate	34	39
	5: Very appropriate	6	13
Quantity	−2: Very insufficient	3	3
	−1: Insufficient	30	18
	0: Adequate	60	70
	1: Excessive	6	9
	2: Very excessive	1	0
Overall assessment	1: Worst	5	4
	2	30	14
	3	28	33
	4	30	37
	5: Best	8	13

### 3.4 ChatGPT 3.5 vs. 4.0

Table 4 shows the results of the comparison of mean scores for “quality, quantity, and overall assessment” between ChatGPT 3.5 and 4.0. A significant difference was observed in all three categories. The answers generated by ChatGPT 4.0 received higher scores in terms of quality and overall assessment, indicating better performance compared to ChatGPT 3.5. For quantity, ChatGPT 4.0 was perceived to be closer to an adequate level compared to ChatGPT 3.5, which was rated as insufficient. For each of the three criteria, ChatGPT 4.0 consistently outperformed ChatGPT 3.5, receiving higher scores in terms of quality and overall assessment.

**Table 4:** Comparison of response evaluations according to version of ChatGPT.

	ChatGPT 3.5	ChatGPT 4.0	*p*-value
Quality	3.40 (0.20)	3.73 (0.34)	<0.01
Quantity	−0.31 (0.18)	0.03 (0.30)	<0.01
Overall assessment	3.21 (0.27)	3.67 (0.40)	<0.01

Quality (5-point Likert scale; 1 very inappropriate, 2 inappropriate, 3 average, 4 appropriate, 5 very appropriate). Quantity (5 Likert; −2 very insufficient, −1 insufficient, 0 adequate, 1 excessive, 2 very excessive). Overall assessment (5 Likert; 1 should not be an answer to 5 perfect answer).

## 4 Discussion

In this study, we sought to evaluate the reliability of medical information related to anesthetic procedures provided by the AI language model chatbot, ChatGPT and explored potential differences between the 3.5 and 4.0 versions. By assessing responses to 30 questions, we observed that both versions of ChatGPT consistently offered reasonably accurate medical information, scoring above the midpoint in terms of quality. Notably, the 4.0 version demonstrated a higher percentage of appropriate or very appropriate responses, reaching 64%, indicating a greater reliability of medical information compared to the 3.5 version. Regarding the quantity of information, both versions were generally perceived as providing an adequate amount of information.

Reviewing the literature, ChatGPT, as a large language model LLM, leverages extensive datasets and advanced machine learning algorithms to facilitate human-like conversations, understanding and responding to complex questions in natural language. These conversations can range from light-hearted topics to scientific discussions (10). The initial version, GPT-1, had 117 million parameters, but in the latest versions, GPT-3.5 and 4.0, the number of parameters has significantly increased, enabling more accurate and human-like responses. The application scope of ChatGPT has expanded to various fields, including healthcare (11). Interest in ChatGPT is growing rapidly. Within a short period of 6 months, there has been a significant increase in published papers about ChatGPT, with 533 produced (12). Among these, the most researched topics are those evaluating ChatGPT’s ability to provide accurate answers and its depth of knowledge (11). ChatGPT is breaking down barriers to universal access to healthcare information, assisting in communication between doctors and patients, and providing standardized, evidence-based information, leading to significant growth in the healthcare domain (13). The role of doctors in understanding and addressing complex health issues for patients and communities is expanding, and new technologies like ChatGPT can effectively support this (14). However, despite already demonstrating impressive capabilities in natural language understanding and generation, various potential applications in the medical field, such as data extraction and decision-making in surgery, are still in the early stages of development (10, 15).

Our study contributes to the ongoing previous studies on the appropriateness of integrating generative AI into the field of medicine. Indeed, the integration of generative AI into medicine is a heavily researched area (16). The medical field accounted for the highest proportion of total publications related to ChatGPT research (11). ChatGPT distinguishes itself with remarkable proficiency in understanding and generating text, attracting attention for its versatile applications (16, 17), extending from medical education to the dissemination of patient information (18–20). Active efforts are underway to deploy ChatGPT across various domains, from crafting medical papers to educational contexts, with high expectations for potential to supplement, if not partially to replace, the role of medical experts (8, 9). However, a recent systematic review underscores persisting challenges with issues related to accuracy, authorship, and bias (20). While prior studies predominantly focused on the model’s utility in assisting medical experts, our study takes a unique perspective by exploring its potential benefits for the general population seeking precise information on anesthetic procedures.

In this study, the questions input into ChatGPT were carefully selected to ensure the potential for generalization. From numerous questions, we condensed them to 30 by excluding similar ones. For instance, instead of asking all the following: (1) What is general anesthesia? What is spinal anesthesia? What is epidural anesthesia? (2) What are the side effects of general anesthesia? What are the side effects of spinal anesthesia? What are the side effects of epidural anesthesia? (3) When is general anesthesia used? When is spinal anesthesia used? When is epidural anesthesia used?,” we selected one type of anesthesia for each of the questions in (1), (2), and (3). ChatGPT’s responses are typically lengthy, and evaluators need to assess both versions of the responses, which means they must read a substantial amount of text. Concerned that increased evaluator fatigue could lead to inaccurate assessments, we considered this factor when determining the number of questions.

In studies assessing the reliability of ChatGPT in providing medical information, the model demonstrated accuracy in general medicine (21). Specific areas, such as cardiovascular disease or liver cirrhosis, also received adequate information (22, 23). Our study stands out among the studies evaluating the reliability of ChatGPT in providing medical information with a substantial number of evaluators. We recruited 31 anesthesiologists for response evaluation to minimize bias and enhance the reliability. Our evaluators, comprising medical staff from major university hospitals in Korea, ensured a comprehensive assessment based on the latest medical knowledge.

Another strength of this study is discerning the superiority of responses based on the model’s version. Our results revealed that version 4.0 consistently demonstrated significantly higher scores than version 3.5 across all evaluation criteria (quality, quantity, and overall score). This was particularly evident within the specific categories analyzed. According to OpenAI, version 4.0 excels in understanding natural language and generating creative responses in complex scenarios, but its application to providing medical information, especially in comparison to the previous version, is not fully guaranteed.^1^ The exceptions noted in 3 out of 30 questions emphasize the need for continued scrutiny and improvement in the model’s reliability.

An easy access to medical information for patients enables informed decisions, potentially minimizing various side effects. AI chatbots are expected to contribute to reducing unnecessary costs in the healthcare system by improving efficiency and reducing the need for additional consultations with doctors. However, the lowest scores categorized as “very inappropriate,” “very insufficient,” and “unable to provide as a response” accounted for 1–2% of the total evaluations. Although this is a small percentage, it does suggest that there is a potential risk that AI chatbots could provide completely incorrect medical information. This issue was similarly raised in other studies related to surgery, where AI provided mostly comprehensive answers (24). Concerns have been highlighted regarding ChatGPT’s potential to deliver dangerously inaccurate information due to shortcomings in situational awareness and consistency (25). While ChatGPT exhibits promise in assisting and informing medical staff, it does not currently appear to be a complete replacement for medical professionals. Future research should focus on presenting scenarios rather than simple questions to evaluate the AI’s ability to generate contextually appropriate responses and to assess the adequacy of the contextual information provided.

When considering whether AI chatbots can partially replace the role of healthcare professionals, besides the accuracy of the information provided, another crucial point of discussion is ethical issues. ChatGPT can collect and store personal health information while interacting with patients, potentially including sensitive medical data. Ensuring data security, monitoring, and implementing robust security measures would be essential for ChatGPT and healthcare institutions (26). The allocation of responsibility for the provided medical information is also important. Currently, there is a lack of supervision and standardization of the responsibility system for ChatGPT. It would be important to establish and apply relevant ethical regulations quickly, in addition to detailed verification of the appropriateness of the information, to ensure the safe use of ChatGPT (27). When such protective measures are in place, the use of AI chatbot in the medical field can be meaningful.

### 4.1 Limitations of the study

There are several limitations in our study. First, despite a wide spectrum of surgery and anesthesia, our investigation focused on a specific subset of questions and the 30 questions we addressed may not cover the full range of potential patient inquiries. To comprehensively evaluate ChatGPT’s medical knowledge in the expansive field of surgery and anesthesia, a more diverse set of questions might be necessary. Second, the 31 reviewers who conducted the evaluations were aware that they were assessing responses from ChatGPT, introducing a potential source of bias. This awareness could have influenced their scoring, making them more lenient or strict in their assessments. Future research should minimize bias by using blind evaluations that do not reveal the source of responses to evaluators. Third, comparison between human responses and ChatGPT’s responses was not conducted to determine whether the AI chatbot could replace humans. However, it’s important to note that when making such comparisons, variations in responses may be influenced by the level or expertise of the human respondents. Fourth, it is crucial to acknowledge that ChatGPT was not explicitly designed for medical purposes. This raises valid concerns about the reference sources for its responses and its coverage of the broad field of medicine. The model’s general-purpose nature may limit its accuracy and relevance when providing medical information. Lastly, our study was conducted exclusively in English, and the applicability of our findings to different countries or linguistic contexts remains uncertain. The cultural and linguistic nuances inherent in medical information may vary across regions. Therefore, to enhance the generalizability of ChatGPT’s performance, further comparative studies conducted in other languages are imperative. In the future, further research should be conducted on how much the patient’s anxiety is reduced and how much the patient’s information demand is satisfied by the medical information provided by ChatGPT.

## 5 Conclusion

Responses regarding anesthetic procedures generated by ChatGPT were overall appropriate, providing a somewhat insufficient to an average amount of information. Notably, responses from the latest version, 4.0, were deemed more accurate compared to the earlier version, 3.5. Moving forward, it is imperative to channel future efforts toward the development and enhancement of research models specifically designed to rigorously evaluate the utility of medical information delivered by AI chatbots.

## Data availability statement

The original contributions presented in the study are included in the article/Supplementary material, further inquiries can be directed to the corresponding authors.

## Author contributions

JC: Writing – original draft. AO: Writing – original draft. JP: Data curation, Methodology, Writing – review & editing. RK: Data curation, Writing – review & editing. SY: Writing – review & editing. DL: Data curation, Writing – review & editing. KY: Supervision, Writing – review & editing.

## Footnotes

^1^ https://openai.com/
