# Supplementary material for: Evaluation of the quality and quantity of artificial intelligence-generated responses about anesthesia and surgery: using ChatGPT 3.5 and 4.0
Source: Front Med (Lausanne). 2024 Jul 11;11:1400153. doi: 10.3389/fmed.2024.1400153 (PMC11269144; doi:10.3389/fmed.2024.1400153)
Supplement: Supplementary file 1 [file Table_1.DOCX]

Supplementary Table 1. Questions and ChatGPT generating answers

| No. | Questions | ChatGPT 3.5 | ChatGPT 4.0 |
| --- | --- | --- | --- |
| 1 | What is general anesthesia and how does it work? | General anesthesia is a medical technique that induces a state of unconsciousness and loss of sensation throughout the body, allowing medical procedures to be performed without causing pain or discomfort to the patient. General anesthesia can be administered through various methods such as inhalation, injection, or a combination of both.  The exact mechanism of action of general anesthesia is not fully understood, but it is believed to affect the central nervous system by altering the normal activity of certain neurotransmitters in the brain. General anesthesia drugs typically work by enhancing the activity of inhibitory neurotransmitters, such as gamma-aminobutyric acid (GABA), or by reducing the activity of excitatory neurotransmitters, such as glutamate.  These changes in neurotransmitter activity lead to a depression of the central nervous system, resulting in loss of consciousness, amnesia, and a lack of sensation throughout the body. General anesthesia also causes a number of physiological changes such as a decrease in blood pressure, heart rate, and respiratory rate, which need to be closely monitored and managed by trained medical professionals during the procedure.  Once the procedure is complete, the anesthesia is gradually stopped, allowing the patient to regain consciousness and return to a normal level of sensation and function. | General anesthesia is a medically induced state of unconsciousness, during which the patient is completely unaware of their surroundings and does not feel pain. It is used for various surgical procedures to ensure patient comfort and enable surgeons to perform complex operations without causing pain or distress.  General anesthesia works by administering a combination of medications, usually through intravenous (IV) injection and inhaled gases, to achieve the desired effects. The anesthetic agents used in general anesthesia can be classified into two main categories:  1. Intravenous anesthetics: These medications are administered through an IV line and quickly induce a state of unconsciousness. Common intravenous anesthetics include propofol, etomidate, and barbiturates like thiopental.  2. Inhaled anesthetics: These are gases or vapors that the patient breathes in through a mask or an endotracheal tube. Common inhaled anesthetics include isoflurane, sevoflurane, and desflurane.  The medications used in general anesthesia affect the central nervous system, specifically targeting the brain and spinal cord. They disrupt the transmission of nerve signals, leading to a loss of sensation, muscle relaxation, amnesia, and unconsciousness. The precise mechanisms through which these drugs induce anesthesia are complex and not yet fully understood.  Anesthesia providers, such as anesthesiologists or nurse anesthetists, closely monitor and manage the administration of anesthetic medications throughout the surgery. They ensure the patient remains unconscious, pain-free, and stable during the procedure, and carefully manage the patient's emergence from anesthesia after surgery.  In summary, general anesthesia is a medically induced state of unconsciousness that provides pain relief, amnesia, and muscle relaxation for patients undergoing surgery. It works by administering a combination of intravenous and inhaled anesthetic agents that act on the central nervous system to achieve the desired effects. |
| 2 | What are the possible complications after general anesthesia? | While general anesthesia is generally considered safe, there are potential complications and side effects that can occur after the procedure. These can vary depending on the patient's individual medical history and the type and length of the surgery performed. Here are some possible complications that can occur after general anesthesia:  1. Nausea and vomiting: This is a common side effect of anesthesia and can occur immediately after the procedure or up to a few days later.    2. Sore throat: Breathing tube used during the procedure can cause a sore throat that usually goes away within a few days.   3. Confusion: Some patients may experience confusion, memory loss, or disorientation after waking up from anesthesia. This is more common in elderly patients or those with pre-existing cognitive issues.   4. Allergic reactions: Rarely, patients can have an allergic reaction to the anesthesia drugs.   5. Respiratory problems: In some cases, anesthesia can cause breathing problems, especially in patients with pre-existing lung conditions.   6. Cardiovascular problems: Anesthesia can affect the cardiovascular system, leading to changes in blood pressure, heart rate, and rhythm.   7. Postoperative pain: Patients may experience pain or discomfort after the procedure, which can be managed with pain medication.   8. Infection: There is a risk of infection at the surgical site, which can lead to fever, redness, swelling, and drainage.   9. Blood clots: Patients who are immobilized for a long time during the procedure are at risk of developing blood clots in their legs, which can potentially travel to the lungs and cause a pulmonary embolism.  It is important for patients to closely follow any postoperative instructions provided by their healthcare provider and report any unusual symptoms or complications immediately. | General anesthesia is typically safe, but as with any medical procedure, there can be some potential complications. These complications can be categorized into short-term and long-term risks.  Short-term complications: 1. Allergic reactions: Some patients may have an allergic reaction to the anesthesia medications, which can cause symptoms ranging from mild to severe.  2. Nausea and vomiting: Some patients may experience nausea and vomiting after anesthesia, which can usually be managed with medications.  3. Sore throat: A sore throat can result from the breathing tube used during the procedure, but it typically resolves within a few days.  4. Dental damage: Occasionally, dental damage may occur from the insertion or removal of the breathing tube. Difficulty urinating: Some patients may have temporary difficulty urinating after surgery, which can usually be managed with time and medications.  5. Postoperative confusion or delirium: This is more common in elderly patients, and it usually resolves within a few days.  6. Hypoxia: Low oxygen levels in the blood can occur, usually as a result of airway problems, requiring prompt intervention by the anesthesiologist.  7. Hypotension: Low blood pressure may develop during anesthesia, which is typically managed by adjusting the anesthetic agents and administering fluids or medications.  8. Aspiration: If stomach contents enter the lungs, it can lead to a serious lung infection called aspiration pneumonia. Malignant hyperthermia: This is a rare but potentially life-threatening reaction to certain anesthesia drugs, characterized by a rapid increase in body temperature and muscle rigidity.   Long-term complications: 1. Postoperative cognitive dysfunction (POCD): Some patients, particularly the elderly, may experience cognitive decline or memory issues after surgery. This can last for weeks or months but usually improves over time.  2. Nerve damage: Rarely, nerve damage may occur from the positioning of the patient during surgery or from direct injury by the surgical instruments. This can result in pain, numbness, or muscle weakness. 3. Respiratory complications: Patients with pre-existing lung conditions may be at higher risk for complications such as pneumonia or respiratory failure.  4. Cardiovascular complications: Those with pre-existing heart problems may be at increased risk for complications such as heart attack, stroke, or blood clots.   It's essential to discuss any concerns with your healthcare provider before undergoing surgery with general anesthesia. Anesthesia providers will take necessary precautions to minimize the risk of complications and monitor you closely during and after the procedure. |
| 3 | What is the mortality rate from anesthesia in healthy people? | The mortality rate from anesthesia in healthy people is very low, with estimates ranging from 0.05 to 0.1% (or 1 in 1,000 to 2,000 cases) according to some studies. However, the risk of complications and mortality can increase in patients with pre-existing medical conditions, such as cardiovascular disease, lung disease, or obesity. The risk also depends on the type of surgery and the duration of anesthesia, as longer and more complex surgeries carry a higher risk of complications.  It's important to note that modern anesthesia techniques and monitoring have significantly improved the safety of general anesthesia in recent years. Anesthesiologists and other healthcare providers use a range of tools and techniques to monitor patients' vital signs and adjust the anesthesia dosage as needed to ensure a safe and successful procedure.  Patients can help minimize the risk of complications by providing a comprehensive medical history to their healthcare provider, following pre-operative instructions carefully, and communicating openly with the anesthesia team about any concerns or questions they may have. | The mortality rate from anesthesia in healthy people is very low. Advances in anesthesia techniques, drugs, and monitoring equipment have significantly reduced anesthesia-related mortality over the past few decades.  For healthy individuals without significant medical issues (classified as ASA I or ASA II according to the American Society of Anesthesiologists' classification system), the mortality rate due to anesthesia alone is estimated to be around 1 in 100,000 to 1 in 200,000 cases. This means that the risk of dying from anesthesia for a healthy person is extremely small.  However, it is essential to recognize that the overall risk of complications or death during surgery is influenced not only by the anesthesia but also by the type of surgery, the patient's overall health, and other factors. To minimize risks, it is crucial to have a thorough preoperative evaluation and discuss any concerns with your anesthesiologist and surgeon. |
| 4 | What is "awakening under anesthesia"? | "Awakening under anesthesia" refers to a rare phenomenon where a patient regains consciousness during surgery or while still under the effects of anesthesia. Although general anesthesia is designed to induce unconsciousness and prevent pain and movement during surgery, some patients may regain consciousness to some degree during the procedure.  The experience of awakening under anesthesia can vary widely among patients, and some may not recall the event at all due to the effects of the drugs on memory. However, some patients may experience distressing symptoms such as awareness of pain or paralysis, feeling trapped or suffocated, or hearing or sensing things happening in the operating room.  Awakening under anesthesia is generally considered a rare occurrence, with reported rates ranging from 0.1% to 0.2% of cases. However, the risk may be higher in certain patient populations, such as those with a history of drug or alcohol abuse, sleep apnea, or obesity.  To minimize the risk of awakening under anesthesia, anesthesiologists may use monitoring techniques such as electroencephalography (EEG) to assess the patient's level of consciousness during the procedure. Additionally, patients who are at higher risk of awareness under anesthesia may be given additional sedative medications or alternative anesthesia techniques to reduce the risk of consciousness. | "Awakening under anesthesia," also known as intraoperative awareness or anesthesia awareness, is a rare event where a patient regains consciousness during surgery while under general anesthesia. This means that the patient becomes aware of their surroundings, can potentially feel pain or discomfort, and might even hear sounds or conversations in the operating room.  Anesthesia awareness can occur due to various factors, such as inadequate anesthesia administration, rapid metabolism of the anesthetic drugs, or errors in monitoring or equipment. The experience can range from a vague awareness of the surroundings to severe distress and pain.  It is important to note that anesthesia awareness is uncommon. The incidence varies in the literature but is generally estimated to occur in around 0.1% to 0.2% of patients undergoing general anesthesia. The risk of anesthesia awareness can be higher in certain situations, such as emergency surgeries, cesarean sections under general anesthesia, or when the use of muscle relaxants makes it challenging to assess the depth of anesthesia accurately.  To minimize the risk of awakening under anesthesia, anesthesiologists closely monitor patients' vital signs, depth of anesthesia, and other parameters during surgery. They use this information to adjust the anesthetic drugs as needed to ensure the patient remains safely unconscious throughout the procedure. If you have concerns about anesthesia awareness, discuss them with your anesthesiologist before your surgery. |
| 5 | What are the side effects of spinal anesthesia? | Like any medical procedure, spinal anesthesia can have side effects and potential complications. Here are some of the most common side effects associated with spinal anesthesia:  1. Low blood pressure: Spinal anesthesia can cause a sudden drop in blood pressure, which may result in dizziness, nausea, or fainting. To minimize this risk, patients are often given intravenous fluids and medication to maintain blood pressure during the procedure.  2. Headache: Spinal anesthesia can cause a headache that typically develops within a few days of the procedure. The headache may be dull or throbbing and may be accompanied by nausea, vomiting, or dizziness. The headache can usually be treated with rest, fluids, and medication.  3. Numbness or tingling: Spinal anesthesia can cause temporary numbness or tingling in the legs or lower body. This is a normal side effect of the medication and typically resolves within a few hours after the procedure.  4. Infection: Like any invasive medical procedure, spinal anesthesia carries a risk of infection. To minimize this risk, the anesthesiologist will use sterile equipment and follow strict infection control procedures.  5. Allergic reaction: In rare cases, patients may experience an allergic reaction to the medication used in spinal anesthesia, which can cause symptoms such as hives, difficulty breathing, or swelling.  6. Back pain: Spinal anesthesia can cause temporary back pain or discomfort at the site of the injection. This typically resolves within a few days and can be managed with rest, ice, and medication.  It's important for patients to discuss the potential risks and side effects of spinal anesthesia with their healthcare provider and anesthesiologist before the procedure. By understanding the potential risks and taking necessary precautions, patients can help minimize the risk of complications and ensure a safe and successful surgical experience. | Spinal anesthesia is generally safe, but as with any medical procedure, there can be potential side effects and complications. Some of the common side effects of spinal anesthesia include:  1. Low blood pressure (hypotension): Spinal anesthesia can cause blood vessels to dilate, leading to a decrease in blood pressure. This is usually managed with intravenous fluids and medications as needed.  2. Headache: Post-dural puncture headache (PDPH) is a potential side effect that occurs due to leakage of cerebrospinal fluid (CSF) through the puncture site. This type of headache typically develops within a few days of the procedure and may be worsened by sitting or standing. It can be treated with hydration, pain relievers, and in some cases, an epidural blood patch.  3. Nausea and vomiting: Although less common than with general anesthesia, some patients may experience nausea or vomiting after spinal anesthesia. 4. Itching (pruritus): This can occur as a side effect of the opioids that are sometimes included in the anesthetic mixture to provide pain relief. Antihistamines or other medications can be used to alleviate itching if needed.  5. Urinary retention: Temporary difficulty in emptying the bladder can occur due to the numbing effect of the spinal anesthesia on the nerves controlling bladder function. This usually resolves as the anesthesia wears off, but in some cases, temporary catheterization may be required.  6. Back pain: Mild discomfort or pain at the injection site is relatively common but usually resolves within a few days.  7. Transient neurological symptoms: Some patients may experience temporary numbness, tingling, or mild weakness in the legs after spinal anesthesia. These symptoms usually resolve within a few days. Rare but more serious complications of spinal anesthesia include:  1. Infection: In rare cases, infection can occur at the injection site or within the spinal canal, potentially leading to meningitis or an epidural abscess. 2. Nerve damage: While uncommon, direct needle injury to the nerves, hematoma formation, or prolonged compression from local anesthetic can lead to nerve damage.  3. High spinal block: In rare cases, the local anesthetic may spread higher than intended within the spinal canal, affecting the nerves responsible for breathing and causing difficulty breathing or other severe symptoms.  4. Allergic reactions: Allergic reactions to the local anesthetic or other medications used during the procedure can occur, ranging from mild rashes to severe anaphylaxis. Your anesthesiologist will closely monitor you throughout the procedure and take appropriate measures to prevent or manage any side effects or complications that may arise. If you have concerns about spinal anesthesia, discuss them with your anesthesiologist and surgeon before the procedure. They can provide additional information and help you make an informed decision about the best anesthesia option for you. |
| 6 | How long does it take for sensation to return after spinal anesthesia? | The duration of spinal anesthesia and the time it takes for sensation to return will vary depending on the individual patient, the type of surgery, and the dose of the anesthetic medication used. However, most patients can expect the effects of spinal anesthesia to last for several hours.  After the surgery, the patient will be monitored closely by healthcare providers, who will assess the return of sensation and movement in the lower half of the body. In general, it can take several hours for sensation and movement to fully return after spinal anesthesia. The time it takes for sensation to return can vary from person to person, but it typically takes longer for motor function to return than for sensory function.  Patients should follow any post-operative instructions provided by their healthcare provider or anesthesiologist to aid in the recovery process. This may include avoiding certain activities or medications, performing specific exercises, and monitoring for signs of complications such as infection or bleeding.  If the patient experiences any unusual or concerning symptoms after spinal anesthesia, such as persistent numbness or weakness in the legs, severe headache, or difficulty urinating, they should contact their healthcare provider or seek medical attention immediately. | The duration of spinal anesthesia and the time it takes for sensation to return after the procedure can vary depending on several factors, such as the type and dose of the local anesthetic used, the patient's individual response to the medication, and the specific surgery or procedure.  In general, it takes approximately 1 to 4 hours for sensation to return after spinal anesthesia. During this time, the patient will gradually regain sensation and motor function in the affected area, starting with a tingling sensation and progressing to full movement and feeling. It's important to note that the timeline can vary, and in some cases, it may take longer for sensation and motor function to return completely.  Throughout the recovery period, the patient should be monitored closely for any signs of complications or adverse reactions. Once sensation and motor function have returned, the patient will typically be assessed by their healthcare team to ensure they are safe to be discharged or transferred to a recovery ward, depending on the type of procedure and the patient's specific circumstances. |
| 7 | When is epidural anesthesia performed? | Epidural anesthesia is a type of regional anesthesia that involves injecting local anesthetic medication into the epidural space, a space around the spinal cord, to numb the nerves in the lower back and abdomen. It is commonly used for pain relief during childbirth, as well as for other surgical procedures involving the lower half of the body.  Epidural anesthesia may be performed in the following situations:  1. Childbirth: Epidural anesthesia is commonly used during childbirth to provide pain relief during labor and delivery. The medication is injected into the epidural space, numbing the nerves that transmit pain signals from the uterus and birth canal.  2. Surgery: Epidural anesthesia may be used for surgeries that involve the lower half of the body, such as hip replacement or abdominal surgery. The medication is injected into the epidural space, numbing the nerves that transmit pain signals from the surgical site. 3. Chronic pain: Epidural anesthesia may be used for chronic pain conditions, such as back pain or sciatica, to provide long-term pain relief. Epidural anesthesia is generally considered safe and effective, but like any medical procedure, it can carry some risks and potential complications. To minimize the risk of complications, patients should provide a comprehensive medical history to the anesthesiologist, follow pre-operative instructions, and inform their healthcare provider of any changes in their health status before the procedure. | Epidural anesthesia is a type of regional anesthesia that involves injecting local anesthetics and/or analgesics into the epidural space surrounding the spinal cord. It's commonly used to provide pain relief or numbness for a variety of surgical procedures and medical conditions. Some common situations in which epidural anesthesia is performed include:  1. Labor and childbirth: Epidural anesthesia is frequently used to provide pain relief during labor and delivery, making the process more comfortable for the mother. It can also be used for cesarean sections if general anesthesia is not required.  2. Lower abdominal or pelvic surgeries: Procedures involving the lower abdomen or pelvic region, such as gynecological surgeries, urological procedures, or colorectal surgeries, may use epidural anesthesia to provide pain control during and after the surgery.  3. Lower limb surgeries: Epidural anesthesia can be used for surgeries involving the lower extremities, such as hip or knee replacements and lower extremity vascular procedures.  4. Postoperative pain management: For certain surgeries, an epidural catheter may be placed before the surgery to provide continuous pain relief during the postoperative period. This helps manage pain and can reduce the need for systemic pain medications. 5. Chronic pain management: Epidural anesthesia can be used to treat chronic pain conditions, such as lower back pain, sciatica, or cancer pain, by delivering pain-relieving medications directly to the affected nerves. The decision to use epidural anesthesia depends on various factors, including the patient's overall health, the type of procedure, and the patient's preferences. Potential risks and benefits should be discussed with the anesthesiologist and surgeon to determine if epidural anesthesia is the best choice for a particular situation. |
| 8 | What are some of the underlying conditions that can be dangerous when receiving anesthesia? | There are several underlying conditions that can increase the risk of complications when receiving anesthesia. These conditions may affect the way the body responds to anesthesia, increase the risk of respiratory or cardiovascular problems, or interfere with the clearance of drugs from the body.  Here are some common underlying conditions that can be dangerous when receiving anesthesia:  1. Cardiovascular disease: Patients with a history of heart disease, such as coronary artery disease, heart failure, or arrhythmias, may be at increased risk of cardiovascular complications during anesthesia.  2. Respiratory disease: Patients with chronic obstructive pulmonary disease (COPD), asthma, or other respiratory problems may be at increased risk of breathing problems during anesthesia. 3. Kidney disease: Patients with impaired kidney function may be at increased risk of drug toxicity or other complications during anesthesia.  4. Liver disease: Patients with impaired liver function may be at increased risk of drug toxicity or other complications during anesthesia.  5. Neurological disease: Patients with a history of stroke, epilepsy, or other neurological conditions may be at increased risk of complications during anesthesia.  6. Obesity: Patients who are obese may be at increased risk of breathing problems, cardiovascular complications, and other complications during anesthesia.  7. Diabetes: Patients with diabetes may be at increased risk of cardiovascular complications and other complications during anesthesia.  8. Allergies: Patients with a history of allergies or allergic reactions to anesthesia drugs may be at increased risk of anaphylaxis or other allergic reactions. It's important for patients to provide a comprehensive medical history to the anesthesiologist and inform them of any underlying conditions or medications they are taking before receiving anesthesia. This can help minimize the risk of complications and ensure a safe and successful surgical experience. | Certain underlying medical conditions can increase the risk of complications or adverse reactions during anesthesia. Some of these conditions include:  1. Obstructive sleep apnea: Patients with sleep apnea are at a higher risk of respiratory complications during and after anesthesia, particularly if given sedatives or opioid pain medications. 2. Cardiovascular diseases: Patients with heart conditions, such as coronary artery disease, heart failure, or arrhythmias, are at a higher risk of experiencing cardiac complications during anesthesia and surgery.  3. Respiratory diseases: Chronic lung conditions like asthma, chronic obstructive pulmonary disease (COPD), or severe bronchitis can increase the risk of respiratory complications during and after anesthesia. 4. Obesity: Obesity can make it more difficult to manage airways and breathing during anesthesia and increase the risk of complications such as respiratory depression, airway obstruction, or postoperative infection. 5. Diabetes: Poorly controlled diabetes can increase the risk of infection, delayed wound healing, and complications related to blood sugar fluctuations during and after surgery.  6. Kidney or liver disease: Impaired kidney or liver function can affect the metabolism and clearance of anesthetic drugs, potentially leading to complications or prolonged effects of anesthesia. 7. Allergies: Patients with a history of allergies or previous adverse reactions to anesthesia or related medications are at a higher risk of experiencing an allergic reaction during anesthesia.  8. Neurological disorders: Conditions like epilepsy, multiple sclerosis, or Parkinson's disease may increase the risk of anesthesia-related complications or interact with anesthetic medications. 9. Bleeding disorders or anticoagulant use: Patients with bleeding disorders or those taking blood-thinning medications may be at an increased risk of bleeding complications during or after surgery. 10. Geriatric patients: Older patients may have a higher risk of complications due to age-related physiological changes, reduced organ function, and the presence of multiple medical conditions. It's important to disclose your complete medical history, including any underlying conditions, medications, or previous reactions to anesthesia, to your anesthesiologist and surgeon before undergoing any procedure. They will assess your specific situation and take appropriate measures to minimize the risks associated with anesthesia. |
| 9 | What items should be evaluated before surgery in a patient with reduced mobility? | Before surgery, patients with reduced mobility may require additional evaluation and precautions to help ensure a safe and successful surgical experience. Here are some items that should be evaluated before surgery in a patient with reduced mobility:  1. Mobility aids: Patients with reduced mobility may rely on mobility aids such as wheelchairs or walkers. The healthcare team should evaluate the patient's mobility aids and make any necessary adjustments or accommodations to ensure they are safely used during the surgery and recovery.  2. Positioning: Patients with reduced mobility may require special positioning during the surgery to prevent complications such as pressure ulcers or nerve damage. The healthcare team should evaluate the patient's positioning needs and make any necessary accommodations. 3. Breathing: Patients with reduced mobility may have a higher risk of respiratory complications during and after surgery, such as pneumonia or atelectasis. The healthcare team should evaluate the patient's respiratory status and make any necessary accommodations, such as deep breathing exercises or respiratory therapy.  4. Nutrition: Patients with reduced mobility may have a higher risk of malnutrition or dehydration due to decreased physical activity. The healthcare team should evaluate the patient's nutritional status and make any necessary accommodations, such as providing extra fluids or supplements. 5. Medications: Patients with reduced mobility may be taking medications that can affect anesthesia or increase the risk of complications during and after surgery. The healthcare team should evaluate the patient's medication regimen and adjust as needed to minimize the risk of complications.  6. Assistance: Patients with reduced mobility may require additional assistance during the surgery and recovery period, such as help with personal hygiene or mobility. The healthcare team should evaluate the patient's needs and make any necessary accommodations to ensure their safety and comfort. By evaluating these items and making necessary accommodations, the healthcare team can help ensure a safe and successful surgical experience for patients with reduced mobility. | In patients with reduced mobility, a comprehensive preoperative evaluation is essential to ensure optimal surgical outcomes and minimize potential risks. The evaluation should consider the patient's overall health, the underlying cause of reduced mobility, and any specific concerns related to anesthesia and surgery. Key aspects to evaluate include:  1. Medical history: Obtain a thorough medical history, including the cause of reduced mobility (e.g., neuromuscular disorders, arthritis, spinal cord injury), as well as any other chronic medical conditions such as cardiovascular, respiratory, or metabolic disorders.  2. Physical examination: Conduct a focused physical examination, paying particular attention to the respiratory and cardiovascular systems, as well as the patient's mobility and joint function.  3. Medications: Review the patient's current medications, including prescription and over-the-counter drugs, as well as supplements or herbal remedies, as some of these may interact with anesthetic agents or increase the risk of bleeding during surgery.  4. Allergies: Assess for any known allergies, particularly to medications or anesthesia, which may impact the choice of anesthetic agents or perioperative medications.  5. Functional status: Evaluate the patient's functional status, including their ability to perform activities of daily living, as this can help the surgical team plan for postoperative care, pain management, and rehabilitation.  6. Nutritional status: Assess the patient's nutritional status, as malnutrition can increase the risk of postoperative complications, delayed wound healing, and infection.  7. Pressure ulcers and skin integrity: Examine the patient's skin, particularly over bony prominences, for any signs of pressure ulcers or other skin breakdown, as these can increase the risk of infection and complicate the recovery process.  8. Airway assessment: Evaluate the patient's airway, as reduced mobility may be associated with difficulties in airway management during anesthesia.  9. Venous thromboembolism (VTE) risk: Assess the patient's risk for developing blood clots (deep vein thrombosis or pulmonary embolism) during or after surgery, as reduced mobility can increase this risk. The surgical team may implement prophylactic measures, such as blood-thinning medications or compression stockings, to minimize the risk of VTE.  10. Anesthesia risk assessment: Discuss the patient's specific risks and concerns related to anesthesia with the anesthesiologist, as well as any potential modifications to the anesthesia plan to optimize safety and comfort.  11. Psychosocial assessment: Evaluate the patient's emotional and psychological well-being, as reduced mobility can be associated with anxiety, depression, or other mental health concerns that may impact the patient's surgical experience and recovery. By conducting a thorough preoperative evaluation and addressing any specific concerns, the surgical team can optimize the patient's safety, comfort, and outcomes during and after surgery. |
| 10 | What items should be evaluated before surgery in patients with alcohol dependence? | Before surgery, patients with alcohol dependence should be evaluated to identify any potential risks or challenges that may arise during and after the procedure. Here are some items that should be evaluated:  1. Alcohol use: The patient's alcohol use should be evaluated to determine the severity of their dependence, how long they have been abstinent, and any withdrawal symptoms that may occur during the perioperative period.  2. Medical history: Patients with alcohol dependence may have pre-existing medical conditions such as liver disease, cardiovascular disease, or nutritional deficiencies that can affect their response to anesthesia and increase the risk of complications. Their medical history should be thoroughly evaluated, and appropriate measures should be taken to manage any pre-existing conditions.  3. Medications: Patients with alcohol dependence may be taking medications such as benzodiazepines or opioids that can interact with anesthesia and increase the risk of respiratory depression or other complications. Their medication list should be reviewed, and any necessary adjustments or precautions should be taken.  4. Nutritional status: Patients with alcohol dependence may have nutritional deficiencies that can affect their recovery from surgery and increase the risk of complications. Their nutritional status should be evaluated, and appropriate measures should be taken to optimize their nutritional status before and after surgery.  5. Psychiatric status: Patients with alcohol dependence may have underlying psychiatric conditions such as depression or anxiety that can affect their response to anesthesia and increase the risk of complications. Their psychiatric status should be evaluated, and appropriate measures should be taken to manage any underlying conditions. 6. Social support: Patients with alcohol dependence may benefit from additional social support before and after surgery to help them cope with the stress of the procedure and maintain their recovery from alcohol dependence. By evaluating these items and taking appropriate precautions, healthcare providers can help ensure a safe and successful surgical experience for patients with alcohol dependence. | Patients with alcohol dependence may have an increased risk of complications during and after surgery. A thorough preoperative evaluation and appropriate management of their condition are essential to optimize surgical outcomes. Some important aspects to assess in patients with alcohol dependence include:  1. Medical history: Obtain a detailed medical history, including the extent and duration of alcohol use, previous alcohol withdrawal episodes, and any associated medical conditions, such as liver disease, gastrointestinal issues, or neurological disorders.  2. Physical examination: Perform a comprehensive physical examination to identify signs of alcohol-related organ damage, such as liver enlargement, jaundice, or signs of malnutrition.  3. Laboratory tests: Order appropriate blood tests to assess liver and kidney function, complete blood count, blood clotting profile, and electrolyte levels. Abnormalities in these tests may indicate alcohol-related organ damage or other issues that require further evaluation and management. 4. Psychological assessment: Evaluate the patient's mental health status, as alcohol dependence is often associated with psychiatric disorders such as depression or anxiety. A referral to a mental health professional may be necessary for further assessment and treatment.  5. Alcohol withdrawal risk assessment: Assess the risk of alcohol withdrawal syndrome, a potentially severe condition that can occur when alcohol-dependent individuals suddenly stop or reduce alcohol consumption. The healthcare team may need to initiate withdrawal management, which may include medications such as benzodiazepines, to prevent complications during the perioperative period.  6. Medication review: Review the patient's medications, including any over-the-counter drugs or supplements, to identify potential interactions with anesthetic agents or other perioperative medications.  7. Anesthesia evaluation: Inform the anesthesiologist about the patient's alcohol dependence to plan the most appropriate anesthesia approach and manage potential complications. Alcohol-dependent patients may have an altered response to anesthetic agents, requiring adjustments in dosages or techniques.  8. Nutritional assessment: Alcohol-dependent patients are at risk of malnutrition and vitamin deficiencies, which can affect wound healing and immune function. Assess the patient's nutritional status and consider supplementation or dietary changes if needed.  9. Preoperative abstinence: Encourage patients to abstain from alcohol before surgery, as this can improve liver function, immune response, and overall surgical outcomes.  10. Postoperative care and support: Plan for appropriate postoperative care, including pain management strategies that minimize the use of opioids, as alcohol-dependent patients may be at higher risk for opioid dependence. Arrange for referrals to addiction specialists or support programs to help the patient maintain long-term abstinence and recovery after surgery. By addressing these aspects before surgery, healthcare providers can develop a comprehensive care plan tailored to the needs of patients with alcohol dependence, minimize complications, and promote a successful recovery. |
| 11 | Is it safe for pregnant women to receive general anesthesia? | General anesthesia is generally considered safe for pregnant women when administered by an experienced anesthesiologist and used in appropriate doses. However, as with any medical procedure, general anesthesia carries some risks and potential complications for both the mother and the unborn baby.  The use of general anesthesia during pregnancy is generally limited to situations where it is necessary for the health of the mother or the baby, such as emergency surgery or certain diagnostic procedures. In these cases, the anesthesiologist will take into account the stage of pregnancy, the type and duration of anesthesia, and the potential risks and benefits to both the mother and the unborn baby.  Some potential risks of general anesthesia during pregnancy include:  1. Fetal distress: General anesthesia can affect the baby's oxygen supply and increase the risk of fetal distress or stillbirth.  2. Preterm labor: General anesthesia can increase the risk of preterm labor or premature delivery.  3. Maternal complications: General anesthesia can increase the risk of maternal complications such as high blood pressure, blood loss, or infection. To minimize the risks of general anesthesia during pregnancy, patients should provide a comprehensive medical history to the anesthesiologist and discuss any concerns or questions before the procedure. Additionally, patients should follow pre-operative instructions and inform their healthcare provider of any changes in their health status before the procedure.  Overall, the decision to use general anesthesia during pregnancy should be made on a case-by-case basis, taking into account the individual patient's medical history and the potential risks and benefits to both the mother and the unborn baby. | General anesthesia can be considered relatively safe for pregnant women when necessary, but it is typically reserved for emergencies or situations where other types of anesthesia are not suitable or effective. There are a few considerations and potential risks when using general anesthesia during pregnancy:  1. Maternal risks: General anesthesia can lead to a temporary decrease in blood pressure and uterine blood flow, which may potentially affect the baby. However, modern anesthetic techniques and medications have significantly reduced these risks.  2. Fetal risks: There is a small risk of preterm labor, low birth weight, or fetal distress associated with general anesthesia, but the overall risk is low when appropriate precautions are taken. The potential risks to the fetus should be weighed against the benefits of the surgery or procedure.  3. Medications: Some anesthetic medications can cross the placenta and may have effects on the fetus. Anesthesiologists are trained to select the most appropriate medications and doses to minimize these risks. 4. Timing: The timing of the surgery or procedure can also influence the safety of general anesthesia during pregnancy. In general, elective surgeries should be postponed until after delivery, if possible. If surgery is necessary, the second trimester is often considered the safest time because organ development is mostly complete, and the risk of preterm labor is lower. To ensure the safety of both the mother and the baby, the following steps should be taken:  1. Consultation: A thorough consultation with the surgeon, anesthesiologist, and obstetrician is crucial to discuss the risks and benefits of general anesthesia and the planned procedure. 2. Monitoring: Close monitoring of both the mother and the baby is essential during the procedure, including monitoring the mother's blood pressure, oxygen levels, and the baby's heart rate. 3. Anesthetic technique: The anesthesiologist will choose the most appropriate anesthetic medications and techniques to minimize risks to both the mother and the baby. 4. Postoperative care: Proper postoperative care, including pain management and monitoring for any complications, is essential for a smooth recovery. If you are pregnant and require surgery or a procedure, it is essential to discuss the risks and benefits of general anesthesia with your healthcare team. They will help you make the best decision based on your specific situation and medical history. |
| 12 | Why do I need to fast before surgery? | Fasting before surgery is necessary to reduce the risk of complications associated with anesthesia. Here are some reasons why you may be required to fast before surgery:  1. To prevent aspiration: When you eat or drink, the contents of your stomach can enter your lungs if you vomit during anesthesia. This can cause a serious complication called aspiration pneumonia, which can be life-threatening. Fasting before surgery helps to ensure that your stomach is empty and reduces the risk of aspiration.  2. To improve anesthesia effectiveness: Some medications used during anesthesia can interact with food or drink, reducing their effectiveness. Fasting before surgery helps to ensure that the medications are absorbed and work properly. 3. To reduce the risk of nausea and vomiting: Surgery and anesthesia can cause nausea and vomiting. Fasting before surgery can help reduce the likelihood of these symptoms. 4. To reduce the risk of complications: Eating or drinking before surgery can increase the risk of complications such as delayed wound healing, infection, or blood clots. The length of time you are required to fast before surgery will depend on several factors, including the type of procedure, the anesthesia type, and your overall health status. Your healthcare provider or anesthesiologist will provide specific instructions regarding fasting before surgery to ensure that you are properly prepared for the procedure and to minimize the risk of complications. | Fasting before surgery is important to ensure your safety during anesthesia. The primary reason for fasting is to reduce the risk of aspiration, which occurs when stomach contents are regurgitated into the throat and then accidentally inhaled into the lungs. Aspiration can lead to serious complications, such as pneumonia or lung damage.  When you are under general anesthesia or sedation, your normal gag reflex and protective airway mechanisms are suppressed, which increases the risk of aspiration. Fasting helps ensure that your stomach is empty, minimizing the chance of stomach contents entering your lungs if you were to regurgitate during anesthesia.  Fasting guidelines may vary depending on the healthcare facility, the type of surgery, and the anesthesia used. Generally, patients are advised to follow these guidelines:  1. Solid foods: You should not consume any solid foods for 6-8 hours before surgery. This allows your stomach enough time to empty its contents.  2. Clear liquids: You may be allowed to consume clear liquids (such as water, clear juices, or black coffee without cream or sugar) up to 2 hours before surgery. Clear liquids empty from the stomach more quickly than solid foods.  3. Non-clear liquids and medications: Your healthcare team may provide specific instructions regarding non-clear liquids (such as milk, cream, or juices with pulp) and medications. In some cases, certain medications may be taken with a small sip of water even during the fasting period, but always follow your healthcare team's guidance. It is essential to follow your healthcare team's fasting instructions closely. If you do not follow the fasting guidelines, your surgery may be delayed or rescheduled to ensure your safety during anesthesia. If you have any questions or concerns about fasting before surgery, consult your healthcare team for clarification. |
| 13 | How long do I need to fast before surgery? | The length of time you need to fast before surgery depends on several factors, including the type of surgery, the type of anesthesia, and your individual health status. In general, patients are typically advised to avoid eating or drinking anything for at least 6 to 8 hours before surgery.  Here are some general guidelines for fasting before surgery:  1. Solid foods: You should avoid eating solid foods for at least 6 hours before surgery. This includes any food that requires chewing, such as meat, bread, vegetables, or fruit.  2. Clear liquids: You may be allowed to drink clear liquids, such as water, clear tea, or black coffee, up to 2 hours before surgery. However, you should check with your healthcare provider and anesthesiologist to confirm whether this is allowed for your specific procedure. 3. Milk and other liquids: You should avoid drinking milk, juices with pulp, or other liquids that contain solids or fats for at least 6 hours before surgery. It's important to follow the fasting instructions provided by your healthcare provider and anesthesiologist to help minimize the risk of complications and ensure a safe and successful surgical experience. If you have any questions or concerns about fasting before surgery, be sure to discuss them with your healthcare provider. | The duration of fasting before surgery is intended to minimize the risk of aspiration (inhaling stomach contents into the lungs) during anesthesia, which can cause serious complications. General guidelines for preoperative fasting are as follows:  1. Solid foods: You should avoid consuming solid foods for at least 6 hours before surgery. This allows enough time for the stomach to empty and reduces the risk of vomiting and aspiration.  2. Clear liquids: You can usually consume clear liquids, such as water, clear juice without pulp, or black tea/coffee without milk or cream, up to 2 hours before surgery. This helps to maintain hydration without increasing the risk of aspiration.  3. Non-clear liquids, milk, or milk-containing beverages: These should be avoided for at least 6 hours before surgery, as they take longer to leave the stomach and are considered similar to solid foods.  4. Fatty or fried foods: It's best to avoid consuming fatty or fried foods in the last meal before surgery, as these can take longer to digest and increase the risk of complications.  5. Medications: Your healthcare provider will give you specific instructions regarding medications you should or should not take before surgery. Some medications may need to be taken with a small sip of water, while others may need to be discontinued or adjusted. These are general guidelines, and individual recommendations may vary depending on factors like the type of surgery, anesthesia used, and your specific medical conditions. It's important to follow the fasting instructions provided by your healthcare team, as they will tailor their recommendations to your specific needs and circumstances. Always consult your surgeon or anesthesiologist for personalized guidance on preoperative fasting. |
| 14 | Do I need to quit smoking before anesthesia? | Yes, it is generally recommended that patients quit smoking before anesthesia to reduce the risk of complications during and after the procedure. Smoking can affect your body's response to anesthesia, increase the risk of respiratory and cardiovascular complications, and slow down the healing process after surgery.  How long you should stop smoking before anesthesia depends on several factors, including the duration and frequency of your smoking habit, your overall health status, and the type of procedure you will be undergoing. In general, patients are advised to stop smoking for as long as possible before surgery to allow their body time to recover from the effects of smoking.  It's important to discuss your smoking cessation plan with your healthcare provider and anesthesiologist before surgery to determine the best approach for your individual situation. They can provide guidance and support to help you quit smoking and reduce the risk of complications during and after surgery. | It is highly recommended to quit smoking before undergoing anesthesia and surgery. Smoking has several negative effects on your body that can increase the risk of complications during and after surgery. Some of these risks include:  1. Respiratory complications: Smoking damages the respiratory system, impairing lung function and increasing the risk of respiratory complications such as pneumonia, bronchitis, and atelectasis (lung collapse) during and after surgery.  2. Poor wound healing: Smoking reduces blood flow and oxygen delivery to tissues, which can slow down wound healing, increase the risk of infection, and result in poorer surgical outcomes.  3. Cardiovascular issues: Smoking increases the risk of heart-related complications during surgery, such as heart attack or abnormal heart rhythms.  4. Anesthetic complications: Smoking can alter the way anesthetic drugs are metabolized and increase the risk of complications during anesthesia, including difficulty in managing the airway.   5. Blood clot risk: Smoking increases the risk of blood clots (deep vein thrombosis) after surgery, which can potentially lead to life-threatening complications. The longer you can quit smoking before surgery, the better. Ideally, you should quit smoking at least 4-6 weeks before surgery, as this allows your body enough time to start healing and improving lung function. However, even quitting a few days or weeks before surgery can still have some benefits in reducing the risk of complications.  It's crucial to discuss your smoking history with your healthcare team, including your surgeon and anesthesiologist. They can provide guidance and resources to help you quit smoking and optimize your health before surgery. |
| 15 | How long do I need to quit smoking to be safe during surgery? | The length of time you need to quit smoking to be safe during surgery depends on several factors, including the duration and frequency of your smoking habit, your overall health status, and the type of procedure you will be undergoing. However, in general, the longer you quit smoking before surgery, the better your chances of reducing your risk of complications and improving your recovery.  Here are some general guidelines for smoking cessation before surgery:  1. At least 24 hours: Patients should avoid smoking for at least 24 hours before surgery. This can help reduce the risk of respiratory and cardiovascular complications during anesthesia. 2. Longer periods: For patients who smoke heavily or have underlying health conditions, it may be recommended to quit smoking for a longer period before surgery, such as 2-4 weeks.  3. Ideal time: Ideally, patients should quit smoking as soon as they learn they will be undergoing surgery. The longer you quit before surgery, the better your chances of reducing your risk of complications and improving your recovery. It's important to discuss your smoking cessation plan with your healthcare provider and anesthesiologist before surgery to determine the best approach for your individual situation. They can provide guidance and support to help you quit smoking and reduce the risk of complications during and after surgery. | There is no specific time frame that guarantees complete safety during surgery after quitting smoking. However, the longer you abstain from smoking before surgery, the better the chances of reducing potential complications and improving your surgical outcome.  Ideally, quitting smoking at least 4 to 6 weeks before surgery can provide significant benefits, including improved respiratory function, better circulation, enhanced immune response, and more effective anesthesia management. Quitting even a few weeks before surgery can have positive effects on your surgical outcome.  It is important to note that the benefits of quitting smoking continue to increase the longer you abstain. In addition to the immediate benefits for surgery, quitting smoking has numerous long-term health benefits, such as reducing the risk of heart disease, stroke, and various types of cancer.  Discuss your smoking habits and plans to quit with your healthcare provider, surgeon, and anesthesiologist. They can provide guidance, resources, and support to help you quit smoking before your procedure and improve your surgical outcome. |
| 16 | The day before surgery, I had a fever, cough, and phlegm. Can I have general anesthesia? | If you have a fever, cough, and phlegm the day before surgery, it's important to inform your healthcare provider and anesthesiologist immediately. They will evaluate your symptoms and determine whether it's safe to proceed with general anesthesia.  Fever, cough, and phlegm can be signs of an underlying respiratory infection, such as bronchitis or pneumonia, which can increase the risk of respiratory complications during and after anesthesia. Anesthesiologists will carefully evaluate your respiratory function and may recommend postponing the procedure if your symptoms are severe or if there is a risk of complications.  In some cases, if the surgery is urgent and cannot be postponed, the anesthesiologist may adjust the type and dosage of anesthesia to minimize the risk of complications. For example, regional anesthesia, such as epidural anesthesia, may be preferred over general anesthesia for some procedures to minimize the risk of respiratory complications.  It's important to be honest and upfront with your healthcare provider and anesthesiologist about your symptoms and any other health issues you may have. By working together, they can determine the best approach to minimize the risk of complications and ensure a safe and successful surgical experience. | I am not a medical professional, but I can provide you with some general information. If you have a fever, cough, and phlegm the day before surgery, it's important to inform your surgeon and anesthesiologist. They will evaluate your condition and decide whether it's safe for you to undergo general anesthesia and proceed with the surgery.  These symptoms could be indicative of an infection, which may increase the risk of complications during and after surgery. Your healthcare team may decide to postpone your surgery or take additional precautions if they deem it necessary. Always follow your healthcare provider's advice and recommendations in these situations. |
| 17 | I am taking anticoagulants. How long should I stop before surgery? | If you are taking anticoagulant medications, such as warfarin, heparin, or aspirin, your healthcare provider will determine whether you need to stop or adjust your medication before surgery. The timing and duration of stopping anticoagulant medications before surgery will depend on the individual patient and the specific medication being used.  In general, patients who are taking anticoagulants may need to stop taking them for a period of time before surgery to reduce the risk of bleeding during and after the procedure. However, it's important to balance the risk of bleeding against the risk of blood clots, which can be dangerous if left untreated.  Here are some general guidelines for stopping anticoagulants before surgery:  1. Warfarin: Patients taking warfarin may need to stop taking the medication 5-7 days before surgery. The duration of stopping may vary depending on the individual patient's situation and the reason for taking warfarin.  2. Heparin: Patients taking heparin may need to stop taking the medication 24-48 hours before surgery. The duration of stopping may vary depending on the individual patient's situation and the type of heparin being used.  3. Aspirin: Patients taking aspirin may need to stop taking the medication 7-10 days before surgery, depending on the individual patient's situation and the reason for taking aspirin. It's important to follow your healthcare provider's instructions carefully regarding the use of anticoagulant medications before surgery. They can provide guidance on when to stop or adjust your medication to help ensure a safe and successful surgical experience. | If you are taking anticoagulants (blood thinners) before surgery, it's crucial to consult with your healthcare provider, surgeon, and anesthesiologist for specific guidance on when to stop and resume these medications. The timing depends on several factors, including the type of anticoagulant, the reason for taking the medication, and the specific surgery you will undergo.  Here are some general guidelines for stopping common anticoagulants before surgery:  1. Warfarin (Coumadin): Warfarin is typically stopped 5 days before surgery. The exact timing may vary depending on your individual risk factors and the results of your blood tests, specifically your international normalized ratio (INR) levels.  2. Direct oral anticoagulants (DOACs): Rivaroxaban (Xarelto): Usually stopped 24 to 48 hours before surgery, depending on your kidney function and the bleeding risk of the procedure.  - Apixaban (Eliquis): Generally stopped 48 to 72 hours before surgery, depending on your kidney function and the procedure's bleeding risk.  - Dabigatran (Pradaxa): Typically discontinued 24 to 96 hours before surgery, depending on your kidney function and the bleeding risk associated with the procedure.  - Edoxaban (Savaysa): Often stopped 48 to 72 hours before surgery, depending on your kidney function and the procedure's bleeding risk.  3. Heparin: Unfractionated heparin given through an intravenous (IV) infusion can be stopped 4 to 6 hours before surgery, while low molecular weight heparin (e.g., enoxaparin) is usually stopped 12 to 24 hours before surgery. These guidelines are for general reference only. It's essential to consult your healthcare provider for personalized instructions based on your individual circumstances. In some cases, they may recommend "bridging therapy" with a shorter-acting anticoagulant to minimize the risk of blood clots while you are off your regular anticoagulant. Always follow your healthcare provider's specific recommendations and never stop or adjust your medications without their guidance. |
| 18 | What medications do I need to keep taking before surgery until the day of surgery? | Before surgery, it's important to review your current medications with your healthcare provider to determine which medications should be continued until the day of surgery. Some medications may need to be continued to manage chronic medical conditions or to prevent withdrawal symptoms, while others may need to be adjusted or stopped before the procedure.  Here are some medications that may need to be continued until the day of surgery:  1. Medications for chronic medical conditions: If you are taking medications to manage chronic medical conditions, such as high blood pressure, diabetes, or heart disease, it's important to continue taking these medications until the day of surgery unless your healthcare provider instructs otherwise. 2. Steroids: If you are taking steroids to manage a chronic medical condition, such as asthma or arthritis, it's important to continue taking these medications until the day of surgery to prevent withdrawal symptoms.  3. Psychiatric medications: If you are taking medications for a psychiatric condition, such as depression or anxiety, it's important to continue taking these medications until the day of surgery to prevent withdrawal symptoms.  4. Seizure medications: If you are taking medications to manage seizures, it's important to continue taking these medications until the day of surgery to prevent seizures.  5. Thyroid medications: If you are taking medications for a thyroid condition, it's important to continue taking these medications until the day of surgery to maintain normal thyroid function. It's important to discuss your current medications with your healthcare provider and anesthesiologist before surgery. They can provide guidance on which medications to continue taking until the day of surgery to help ensure a safe and successful surgical experience. | The decision to continue or discontinue medications before surgery depends on the specific medication, your medical history, and the type of surgery. Your healthcare provider, surgeon, and anesthesiologist will provide personalized guidance on which medications to keep taking until the day of surgery.  However, some medications that are commonly continued up to and including the day of surgery include:  1. Heart medications: Many heart medications, such as beta-blockers, calcium channel blockers, and angiotensin-converting enzyme (ACE) inhibitors, should generally be continued up to and including the day of surgery, but specific instructions may vary. Your healthcare provider will give you personalized instructions.  2. Asthma medications: Inhalers and other asthma medications are usually continued to ensure optimal respiratory function during surgery.  3. Anti-seizure medications: Antiepileptic medications are typically continued to prevent seizures during surgery and the recovery period.  4. Thyroid medications: Thyroid hormone replacement medications, such as levothyroxine, are usually continued to maintain appropriate hormone levels during surgery.  5. Some blood pressure medications: Many blood pressure medications should be continued, but specific instructions may vary. ACE inhibitors or angiotensin receptor blockers (ARBs) might be withheld on the day of surgery. Your healthcare provider will provide personalized guidance.  6. Selective serotonin reuptake inhibitors (SSRIs) and other psychiatric medications: In most cases, psychiatric medications like SSRIs, SNRIs, or other antidepressants should be continued, as stopping them abruptly can lead to withdrawal symptoms or worsening of psychiatric symptoms. However, some medications, such as monoamine oxidase inhibitors (MAOIs), may need to be stopped before surgery. Consult your healthcare provider for specific instructions. These are general guidelines, and your healthcare provider may have different instructions for you based on your specific medical history, current medications, and the type of surgery. Always consult your healthcare provider before stopping or adjusting any medication, and follow their specific instructions regarding preoperative medication management. |
| 19 | What diseases or conditions require cardiac evaluation before surgery? | Patients with certain diseases or conditions may require cardiac evaluation before surgery to assess their risk of cardiovascular complications during and after the procedure. The need for cardiac evaluation depends on several factors, including the type of surgery, the patient's overall health status, and their individual risk factors.  Here are some diseases or conditions that may require cardiac evaluation before surgery:  1. Coronary artery disease (CAD): Patients with a history of CAD or angina may require cardiac evaluation before surgery to assess their risk of heart attack or other cardiac events during the procedure.  2. Heart failure: Patients with heart failure may require cardiac evaluation before surgery to assess their heart function and risk of cardiac complications during and after the procedure.  3. Arrhythmias: Patients with arrhythmias, such as atrial fibrillation or ventricular tachycardia, may require cardiac evaluation before surgery to assess their risk of developing arrhythmias during the procedure.  4. Valvular heart disease: Patients with valvular heart disease, such as aortic stenosis or mitral regurgitation, may require cardiac evaluation before surgery to assess their heart function and risk of complications during and after the procedure.  5. Pulmonary hypertension: Patients with pulmonary hypertension may require cardiac evaluation before surgery to assess their heart function and risk of complications during and after the procedure.  6. Diabetes: Patients with diabetes may require cardiac evaluation before surgery to assess their risk of cardiovascular complications and to optimize their blood sugar control before the procedure. It's important to discuss your medical history and any underlying health conditions with your healthcare provider and anesthesiologist before surgery to determine if cardiac evaluation is necessary. They can provide guidance on the appropriate testing and management strategies to help reduce your risk of cardiovascular complications during and after surgery. | A cardiac evaluation may be necessary before surgery for patients with certain diseases, conditions, or risk factors to assess their cardiovascular health and determine the risk of potential complications during the procedure. Some situations that may require a cardiac evaluation before surgery include:  1. A history of coronary artery disease or myocardial infarction (heart attack): Patients with a history of heart disease or prior heart attacks may be at higher risk for complications during surgery.  2. Heart failure: Patients with heart failure may require a cardiac evaluation to determine their ability to tolerate the stress of surgery and anesthesia.  3. Significant valvular heart disease: Conditions like aortic stenosis, mitral stenosis, or mitral regurgitation can affect heart function and may require evaluation before surgery.  4. Cardiac arrhythmias: Patients with a history of arrhythmias, particularly atrial fibrillation or ventricular tachycardia, may need a cardiac evaluation to assess the risk of complications during surgery.  5. Pulmonary hypertension: Patients with pulmonary hypertension may require a cardiac evaluation to determine the impact of their condition on heart function and assess the risk of complications during surgery.  6. Peripheral arterial disease: This condition can be associated with an increased risk of coronary artery disease, and patients may require a cardiac evaluation to assess their risk.  7. A history of stroke or transient ischemic attack (TIA): These patients may have an increased risk of cardiovascular disease and may require a cardiac evaluation before surgery.  8. Presence of multiple cardiovascular risk factors: Patients with multiple risk factors, such as diabetes, hypertension, high cholesterol, smoking, obesity, or a family history of heart disease, may require a cardiac evaluation to assess their risk for complications during surgery. 9. Age: Older patients, especially those above 65 years, may require a cardiac evaluation due to an increased likelihood of having undiagnosed cardiovascular disease.  10. Major or high-risk surgeries: Patients undergoing major or high-risk surgeries, such as vascular, thoracic, or abdominal procedures, may require a cardiac evaluation due to the increased stress on the heart during these procedures. It's important to discuss your medical history and any pre-existing conditions with your healthcare provider, surgeon, and anesthesiologist. They will determine whether a cardiac evaluation is necessary and provide guidance on any additional testing or consultations needed to ensure your safety during surgery. |
| 20 | What diseases or conditions require lung-related evaluation before surgery? | Several diseases or conditions may require a lung-related evaluation before surgery to help ensure a safe and successful surgical experience. The need for a lung-related evaluation may depend on the patient's medical history, the type of surgery, and the level of risk for developing respiratory complications during and after the procedure. Here are some conditions that may require a lung-related evaluation before surgery:  1. Chronic obstructive pulmonary disease (COPD): Patients with COPD may require a lung function test before surgery to assess their lung function and determine the risk of respiratory complications during and after the procedure.  2. Asthma: Patients with asthma may require a lung function test before surgery to assess their lung function and determine the risk of respiratory complications during and after the procedure. 3. Smoking history: Patients with a history of smoking may require a lung function test before surgery to assess their lung function and determine the risk of respiratory complications during and after the procedure.  4. Sleep apnea: Patients with sleep apnea may require a sleep study before surgery to assess their breathing during sleep and determine the risk of respiratory complications during and after the procedure.  5. Pulmonary hypertension: Patients with pulmonary hypertension may require a lung function test and cardiac evaluation before surgery to assess their lung and cardiac function and determine the risk of respiratory and cardiac complications during and after the procedure.  6. Interstitial lung disease: Patients with interstitial lung disease may require a lung function test and chest imaging before surgery to assess their lung function and determine the risk of respiratory complications during and after the procedure. It's important to discuss your medical history with your healthcare provider and anesthesiologist before surgery to determine whether a lung-related evaluation is necessary. They can provide guidance on how to manage any underlying medical conditions and reduce the risk of respiratory complications during and after the procedure. | A preoperative lung-related evaluation, also known as a pulmonary evaluation, may be necessary for certain patients to assess the risk of respiratory complications during and after surgery. Factors that can influence the need for a preoperative pulmonary evaluation include the type of surgery, the patient's overall health, and the presence of specific medical conditions.  Diseases or conditions that may require a pulmonary evaluation before surgery include:  1. Chronic obstructive pulmonary disease (COPD): Patients with COPD, especially those with moderate to severe disease or recent exacerbations, may need a pulmonary evaluation before surgery. 2. Asthma: Patients with poorly controlled asthma or a history of severe asthma exacerbations may require a preoperative pulmonary evaluation to optimize their lung function before surgery.  3. Obstructive sleep apnea (OSA): Patients with known or suspected OSA may need a pulmonary evaluation to assess their risk of complications during and after surgery, and to optimize their treatment if needed. 4. Interstitial lung disease (ILD): Patients with ILD, such as pulmonary fibrosis or sarcoidosis, may need a pulmonary evaluation to assess their lung function and risk of complications during surgery. 5. Pulmonary hypertension: Patients with pulmonary hypertension should undergo a pulmonary evaluation to assess their risk of complications during and after surgery.  6. History of lung cancer or lung surgery: Patients with a history of lung cancer, lung surgery, or other lung-related procedures may need a pulmonary evaluation before surgery to assess their lung function and potential risks.  7. Cystic fibrosis: Patients with cystic fibrosis may require a pulmonary evaluation before surgery to assess their lung function, infection status, and overall health. 8. Severe obesity: Patients with severe obesity are at an increased risk of respiratory complications during and after surgery, and may benefit from a pulmonary evaluation.  9. Smoking: Patients with a significant smoking history or current smokers may need a pulmonary evaluation to assess their lung function and risk of complications during surgery. The need for a preoperative pulmonary evaluation depends on the specific patient, their medical history, and the type of surgery they are undergoing. Your healthcare provider will determine if a pulmonary evaluation is necessary based on your individual risk factors and the anticipated stress of the surgical procedure. |
| 21 | What kind of evaluation is needed for hypertensive patients before surgery? | Hypertensive patients may require an evaluation before surgery to assess their cardiac and overall health status and determine the risk of cardiac complications during and after the procedure. The evaluation may include a physical examination, laboratory tests, and imaging studies. Here are some components of the evaluation that may be needed for hypertensive patients before surgery:  1. Blood pressure control: Hypertensive patients should have their blood pressure well controlled before surgery to reduce the risk of cardiac complications. They may need to adjust their medication regimen or change their lifestyle habits to achieve optimal blood pressure control. 2. Electrocardiogram (ECG): An ECG may be performed to assess the electrical activity of the heart and detect any abnormalities that may increase the risk of cardiac complications during and after surgery.  3. Echocardiogram: An echocardiogram may be performed to assess the structure and function of the heart and detect any abnormalities that may increase the risk of cardiac complications during and after surgery.  4. Laboratory tests: Laboratory tests, such as a complete blood count (CBC), electrolyte panel, and kidney function tests, may be performed to assess overall health status and detect any underlying medical conditions that may increase the risk of surgical complications.  5. Imaging studies: Imaging studies, such as a chest X-ray or CT scan, may be performed to assess lung function and detect any abnormalities that may increase the risk of respiratory complications during and after surgery. It's important to discuss your medical history and current medications with your healthcare provider and anesthesiologist before surgery. They can provide guidance on how to manage your blood pressure and reduce the risk of cardiac complications during and after the procedure. | For patients with hypertension (high blood pressure) undergoing surgery, a preoperative evaluation is essential to assess and manage potential risks associated with the procedure. The evaluation typically includes the following steps:  1. Blood pressure measurement: Accurate measurement of blood pressure is crucial. Your healthcare provider will review your recent blood pressure readings to determine if your hypertension is well-controlled or if adjustments in your treatment plan are necessary.  2. Medical history and physical examination: Your healthcare provider will review your medical history, including the duration and severity of your hypertension, your current medications, and any history of complications or end-organ damage related to hypertension (e.g., heart, kidneys, or eyes). A physical examination will be performed to assess your overall health and identify any potential issues related to hypertension. 3. Laboratory tests: Blood tests may be ordered to evaluate your kidney function, electrolyte levels, and other relevant parameters, as hypertension can affect kidney function and electrolyte balance. Urinalysis may also be performed to check for protein in the urine, which can indicate kidney damage.  4. Electrocardiogram (ECG): An ECG may be performed to assess your heart's electrical activity and identify any potential heart-related issues, such as arrhythmias or signs of ischemia, that may be associated with hypertension.  5. Echocardiogram: In some cases, particularly for patients with poorly controlled hypertension or a history of heart-related complications, an echocardiogram may be recommended to evaluate the structure and function of your heart, including the heart valves and the presence of left ventricular hypertrophy.  6. Medication review and adjustment: Your healthcare provider will review your current medications and may make adjustments to optimize your blood pressure control before surgery. Some blood pressure medications, like ACE inhibitors or angiotensin receptor blockers (ARBs), may be withheld on the day of surgery, depending on your specific situation.  7. Blood pressure management plan: Your healthcare provider will develop a blood pressure management plan for the perioperative period, which may include recommendations for adjusting your medications and monitoring your blood pressure during and after surgery. The specific evaluation needed for hypertensive patients before surgery depends on the individual's medical history, the severity and control of their hypertension, and the type of surgery they are undergoing. Your healthcare provider will determine the appropriate evaluation and management plan based on your unique situation. |
| 22 | A patient with a pacemaker is scheduled to receive general anesthesia. What should I watch out for? | When a patient with a pacemaker is scheduled to receive general anesthesia, there are several considerations and precautions that need to be taken to ensure a safe and successful surgical experience. Here are some things to watch out for:  1. Pacemaker function: Before surgery, the pacemaker function should be checked to ensure that it is working properly and that the settings are appropriate for the patient's medical condition.  2. ECG monitoring: During surgery, the patient's heart rhythm should be monitored closely using an electrocardiogram (ECG) to detect any abnormalities or changes in the pacemaker function.  3. Electromagnetic interference (EMI): General anesthesia equipment and surgical instruments can generate electromagnetic interference (EMI) that can interfere with pacemaker function. The anesthesiologist should take precautions to minimize EMI exposure and ensure that the pacemaker is functioning properly.  4. Medications: Some medications used during general anesthesia, such as muscle relaxants, can affect the pacemaker function and may need to be adjusted or avoided in patients with pacemakers.  5. Post-operative monitoring: After surgery, the patient should be monitored closely for any signs of pacemaker dysfunction or cardiac complications. It's important to discuss the patient's pacemaker function and medical history with the healthcare team, including the anesthesiologist, to ensure that appropriate precautions and monitoring are taken during and after surgery. In some cases, additional testing or consultation with a cardiac specialist may be necessary to help ensure a safe and successful surgical experience. | I am not a medical professional, but I can provide you with some general information to consider when a patient with a pacemaker is scheduled to receive general anesthesia. It is essential to consult with a medical professional for specific guidance.  1. Pacemaker type and settings: Obtain information about the type of pacemaker, settings, and any recent adjustments or complications. This information will help the anesthesiologist and surgical team plan for potential interactions or issues during surgery.  2. Preoperative pacemaker evaluation: It is recommended to have the pacemaker checked and evaluated by a cardiologist or a specialized technician before surgery. They can ensure the device is functioning correctly and make any necessary adjustments to minimize risks during surgery.  3. Electrosurgery interference: Electrosurgical devices, such as electrocautery or diathermy, can cause electromagnetic interference (EMI) with pacemakers. The surgical and anesthesia team should be aware of the potential for interference and take precautions, such as using bipolar electrocautery, limiting the energy output, and ensuring the grounding pad is placed as far away from the pacemaker as possible. Magnet application: In some cases, a magnet may be applied over the pacemaker during surgery to temporarily switch it to a fixed pacing mode, which can reduce the risk of interference from electrosurgery. However, this should be done in consultation with the cardiologist or pacemaker specialist. 4. Monitoring: Continuous monitoring of the patient's vital signs, including electrocardiography (ECG), is crucial to detect any changes in heart rate or rhythm and to ensure the pacemaker is functioning correctly during surgery.  5. Anesthesia considerations: Some anesthetic agents may affect the patient's heart rate or the function of the pacemaker. The anesthesiologist should be aware of the patient's pacemaker and choose anesthetic agents accordingly to minimize any potential risks.  6. Postoperative care: After surgery, the pacemaker should be reevaluated by a cardiologist or a specialized technician to ensure its proper functioning and make any necessary adjustments.  7. Communication: Good communication between the surgical team, anesthesia team, cardiologist, and pacemaker specialist is essential to ensure the patient's safety and the best possible outcome during surgery. Always consult with a medical professional for specific guidance related to a patient with a pacemaker receiving general anesthesia. |
| 23 | What kind of evaluation do asthma patients need before surgery? | Asthma patients may require a pre-operative evaluation before surgery to help ensure a safe and successful surgical experience. The evaluation may include a review of the patient's medical history, physical examination, and laboratory tests. The evaluation will help determine the patient's level of risk for developing asthma exacerbations during and after surgery.  Here are some components of a pre-operative evaluation for asthma patients:  1. Medical history: A review of the patient's medical history, including any history of asthma exacerbations, hospitalizations, or use of oral corticosteroids.  2. Physical examination: A physical examination, including lung function tests, peak flow measurements, and assessment of any respiratory symptoms, such as cough, wheezing, or shortness of breath.  3. Asthma control: Assessment of the patient's asthma control, including their use of asthma medications and any recent changes in asthma symptoms. 4. Lung function tests: Pulmonary function tests, including spirometry and bronchodilator reversibility testing, may be ordered to assess the patient's lung function and the severity of their asthma.  5. Chest x-ray: A chest x-ray may be ordered to assess the patient's lung function and detect any signs of respiratory infections or other lung diseases.  6. Asthma action plan: The patient's asthma action plan, which outlines how to manage asthma exacerbations and use of rescue medications, may need to be updated or adjusted for the perioperative period. The results of the pre-operative evaluation will help determine the patient's level of risk for developing asthma exacerbations during and after surgery. Depending on the patient's level of risk, additional measures may be taken to manage their asthma and reduce the risk of exacerbations, such as adjusting their medications, monitoring their lung function more closely during and after surgery, and ensuring access to rescue medications. | Asthma patients may require additional evaluation and management before surgery to minimize the risk of complications during and after the procedure. The evaluation process for asthma patients may include the following steps:  1. Detailed medical history: A thorough review of the patient's medical history, including the severity and frequency of asthma symptoms, previous asthma exacerbations, hospitalizations, medications, and any history of intubation or respiratory failure.  2. Current asthma control: Assessment of the patient's current asthma control, including the frequency of daytime and nighttime symptoms, use of rescue medications, and any limitations in daily activities. This may involve the use of standardized questionnaires or clinical interviews.  3. Pulmonary function tests: Spirometry or other pulmonary function tests can help determine the severity of asthma and the patient's lung function. These tests may be performed in the weeks leading up to the surgery to guide preoperative management and optimize lung function. 4. Collaboration with healthcare professionals: Communication and collaboration with the patient's primary care physician, pulmonologist, or allergist are essential. They can provide valuable input on the patient's current asthma control, medication regimen, and any concerns that may arise during the perioperative period. 5. Medication review: A thorough evaluation of the patient's current medications, including inhaled corticosteroids, long-acting bronchodilators, and rescue inhalers, to ensure optimal asthma control before surgery. This may involve adjusting the dosages or adding medications as needed. 6. Preoperative optimization: Your healthcare team may recommend strategies to improve your asthma control before surgery, such as increasing the use of inhaled corticosteroids, starting a short course of oral corticosteroids, or adjusting other medications.  7. Anesthesia consultation: The anesthesiologist should be informed of the patient's asthma history, medications, and any specific concerns. They can then recommend appropriate anesthetic techniques and medications to minimize the risk of complications, such as bronchospasm, during surgery.  8. Postoperative care planning: Coordination with the surgical and respiratory teams to ensure appropriate postoperative care, including monitoring for signs of asthma exacerbation, providing adequate pain control, and ensuring the patient has access to their rescue inhaler or other asthma medications. These evaluations and interventions can help minimize the risk of complications during surgery for patients with asthma. However, the specific evaluation process may vary depending on the patient's individual needs, the surgical procedure, and the healthcare team's expertise. |
| 24 | What precautions should be taken before anesthesia for people with poor blood sugar control? | Patients with poor blood sugar control may require additional precautions before anesthesia to help ensure a safe and successful surgical experience. Here are some precautions that may be taken:  1. Blood sugar monitoring: The patient's blood sugar levels should be monitored closely before, during, and after the procedure to prevent hypoglycemia (low blood sugar) or hyperglycemia (high blood sugar).  2. Medication management: The patient's medications, including insulin and other blood sugar-lowering medications, may need to be adjusted before the procedure. It's important to follow your healthcare provider's instructions carefully regarding when to take your medications and when to stop them.  3. Fasting: Fasting requirements may need to be adjusted for patients with poor blood sugar control to prevent hypoglycemia. Patients with diabetes should be monitored closely during fasting to prevent complications.  4. Anesthesia plan: An anesthesia plan should be developed that takes into account the patient's blood sugar control and any potential complications related to their diabetes. The anesthesiologist should be aware of the patient's medications and any potential interactions with anesthesia medications.  5. Postoperative monitoring: The patient should be closely monitored after surgery for any signs of blood sugar imbalances, such as hypoglycemia or hyperglycemia. It's important to discuss your medical history with your healthcare provider and anesthesiologist before surgery to determine whether additional precautions are necessary. They can provide guidance on how to manage your blood sugar levels and reduce the risk of complications during and after the procedure. | For people with poor blood sugar control, such as those with diabetes, it is essential to take specific precautions before undergoing anesthesia to minimize risks and complications. Here are some general precautions to consider:  1. Inform your healthcare team: Make sure your healthcare team, including your surgeon and anesthesiologist, is aware of your diabetes and your current blood sugar control status. This information is crucial for proper planning and management during the perioperative period.  2. Blood sugar monitoring: In the days leading up to your surgery, monitor your blood sugar levels closely and follow your healthcare provider's recommendations to maintain optimal control. Your healthcare team may adjust your diabetes medication regimen to keep your blood sugar within a safe range before and after surgery. 3. Preoperative testing: Your healthcare team may order additional tests, such as hemoglobin A1c (HbA1c) or blood glucose levels, to assess your overall blood sugar control and help guide perioperative management.  4. Medication adjustments: Depending on the type of anesthesia and surgery, your healthcare team may instruct you to adjust or temporarily discontinue certain diabetes medications, especially on the day of surgery. Always follow their guidance on which medications to take or hold and when to resume your usual medication regimen.  5. Fasting guidelines: Generally, patients are asked to fast (no food or drink) for several hours before surgery to reduce the risk of aspiration. However, fasting can affect blood sugar levels. Your healthcare team will provide specific instructions on fasting and may recommend adjustments to your medication regimen to prevent hypoglycemia (low blood sugar).  6. Intraoperative management: During surgery, your anesthesiologist will closely monitor your blood sugar levels and administer insulin or glucose as needed to maintain optimal blood sugar control. 7. Postoperative care: After surgery, continue monitoring your blood sugar levels and follow your healthcare provider's guidance regarding medication adjustments and blood sugar control. It's essential to manage pain and nausea, as these can affect blood sugar levels as well. These precautions are general guidelines, and the specific measures taken may vary based on your individual needs, the type of surgery, and the healthcare team's expertise. It's important to maintain open communication with your healthcare team to ensure the best possible outcomes. |
| 25 | What kind of evaluation do patients with psychiatric problems such as anxiety and depression need before surgery? | Patients with psychiatric problems, such as anxiety and depression, may require a pre-operative evaluation before surgery to help ensure a safe and successful surgical experience. The evaluation may include a review of the patient's medical history, psychiatric history, and medication use. The evaluation will help determine the patient's level of risk for developing psychiatric symptoms during and after surgery.  Here are some components of a pre-operative evaluation for patients with anxiety and depression:  1. Medical history: A review of the patient's medical history, including any history of psychiatric disorders, hospitalizations, and medication use.  2. Psychiatric history: A review of the patient's psychiatric history, including any history of anxiety, depression, or other mood disorders, as well as any history of substance abuse or dependence.  3. Medications: The patient's medications should be reviewed, and any necessary adjustments should be made to ensure they are optimally controlled before and after surgery. 4. Anesthesia plan: An anesthesia plan should be developed that takes into account the patient's psychiatric history and the potential for anxiety or depression symptoms during and after surgery. This may include adjustments to the type and dose of anesthesia medications used.  5. Postoperative monitoring: The patient should be closely monitored after surgery for any signs of anxiety or depression symptoms, such as changes in mood, appetite, or sleep patterns. It's important to discuss your medical history with your healthcare provider and anesthesiologist before surgery to determine whether a pre-operative evaluation is necessary. They can provide guidance on how to manage your psychiatric symptoms and reduce the risk of complications during and after the procedure. Additionally, patients with severe anxiety or depression may require additional support or resources, such as counseling or medication, to help manage their symptoms before and after surgery. | Before surgery, patients with psychiatric problems such as anxiety and depression should undergo a thorough preoperative evaluation to ensure the best possible surgical outcome and postoperative recovery. The evaluation may include the following aspects:  1. Medical history: The healthcare team will review the patient's psychiatric history, including any current or past diagnoses, medications, and treatment plans. This information can help the team identify potential risks and develop strategies to manage them during and after surgery. 2. Medication review: A review of the patient's current medications, including any psychotropic drugs, is essential. Some medications may interact with anesthetic agents or increase the risk of complications. The anesthesiologist and surgical team need to be aware of these potential interactions and adjust medications or anesthesia plans accordingly.  3. Mental health professional consultation: In some cases, a consultation with a mental health professional, such as a psychiatrist or psychologist, may be necessary to assess the patient's current mental health status and provide recommendations for perioperative management.  4. Anxiety and depression assessment: Assessing the severity of anxiety and depression symptoms is crucial for determining the appropriate level of support and interventions needed during the perioperative period.  5. Coping strategies: Identifying effective coping strategies and providing patients with resources to help manage anxiety and depression during the surgical process is essential.  6. Communication and collaboration: Clear communication between the patient, the surgical team, and the mental health professionals is vital to ensure a coordinated care plan that addresses the patient's psychiatric needs.  7. Informed consent: The patient should be provided with comprehensive information about the surgical procedure, potential risks, and expected outcomes to make an informed decision about their care. Ultimately, the goal of the preoperative evaluation is to optimize the patient's mental health, minimize perioperative risks, and facilitate a smooth postoperative recovery. Each patient's evaluation will be tailored to their unique needs and circumstances. |
| 26 | What is the most commonly used drug for sedation or anesthesia? | The most commonly used drug for sedation or anesthesia depends on the type of procedure being performed and the patient's medical history. However, in general anesthesia, a combination of drugs is usually used to induce and maintain a state of unconsciousness and analgesia (pain relief) during surgery.  The drugs used in general anesthesia typically include intravenous anesthetics, such as propofol, midazolam, and etomidate, as well as inhalational anesthetics, such as nitrous oxide, desflurane, and sevoflurane.  For sedation, benzodiazepines such as midazolam or lorazepam, and opioids such as fentanyl, may be used. However, the choice of sedative medication depends on the patient's individual needs and the type of procedure being performed.  It's important to note that the selection of medications and doses used in anesthesia and sedation are highly individualized and tailored to the patient's specific medical history and needs. Only a qualified healthcare provider with specialized training in anesthesia should administer these medications. | The most commonly used drugs for sedation or anesthesia can vary depending on the specific context and purpose. However, some widely used agents include:  1. Propofol: A short-acting sedative-hypnotic agent often used for the induction and maintenance of general anesthesia, as well as for sedation during procedures.  2. Midazolam: A benzodiazepine that is commonly used for sedation and anxiolysis before and during procedures.  3. Fentanyl: A potent synthetic opioid often used for pain relief and as an adjunct to anesthesia. These are just a few examples, and there are many other drugs used for sedation and anesthesia depending on the specific needs of the patient and the procedure being performed. An anesthesiologist will carefully select and administer the appropriate drugs for each individual case. |
| 27 | How can I manage pain after surgery? | Managing pain after surgery is an important part of the recovery process. Here are some ways to manage pain after surgery:  1. Medications: Your healthcare provider may prescribe pain medications, such as opioids or non-steroidal anti-inflammatory drugs (NSAIDs), to manage pain after surgery. It's important to take these medications as directed and to be aware of any potential side effects.  2. Ice and heat: Applying ice or heat to the surgical site can help reduce pain and swelling. Ice is generally recommended for the first 24 to 48 hours after surgery, followed by heat therapy.  3. Rest and elevation: Resting and elevating the affected body part can help reduce pain and swelling. It's important to avoid activities that may worsen pain or delay healing.  4. Physical therapy: Depending on the type of surgery, physical therapy may be recommended to help improve mobility and reduce pain.  5. Relaxation techniques: Relaxation techniques, such as deep breathing, meditation, or visualization, can help reduce pain and promote relaxation.  6. Acupuncture: Acupuncture is a complementary therapy that involves the insertion of fine needles into specific points on the body to relieve pain.   It's important to discuss your pain management plan with your healthcare provider before and after surgery to ensure that you are using the most effective and safe methods for managing your pain. | Pain management after surgery is essential for a comfortable and successful recovery. There are various strategies and medications that can help you manage postoperative pain effectively:  1. Medications: Your healthcare team may prescribe a combination of medications to manage pain, including opioids, non-opioid analgesics (acetaminophen, NSAIDs), and adjuvant medications (muscle relaxants, gabapentinoids). It's essential to follow your healthcare provider's instructions regarding dosage and administration. 2. Regional anesthesia: For certain surgeries, regional anesthesia techniques, such as nerve blocks or epidural anesthesia, can provide targeted pain relief for an extended period after the surgery.  3. Cold therapy: Applying cold packs to the surgical area can help reduce inflammation and provide pain relief. Be sure to wrap the cold pack in a cloth or towel to avoid direct contact with the skin, and apply it for 15-20 minutes at a time, with breaks in between.  4. Heat therapy: For some types of pain, applying a warm compress or heating pad can help alleviate discomfort. Always follow your healthcare provider's recommendations and avoid direct contact with the skin.  5. Elevate the affected area: If possible, elevate the surgical site to help reduce swelling and discomfort.  6. Gentle movement and exercise: As recommended by your healthcare provider, engage in gentle movement and light exercise, like walking, to help improve circulation and promote healing. Avoid strenuous activity or any movements that exacerbate your pain.  7. Rest: Ensure you get enough rest and sleep to allow your body to heal. Follow your healthcare provider's instructions regarding activity levels and wound care.  8. Relaxation techniques: Practice relaxation techniques like deep breathing, meditation, or guided imagery to help manage pain and reduce stress.  9. Compression garments: For some surgeries, your healthcare provider may recommend wearing compression garments to reduce swelling and discomfort. It's important to communicate openly with your healthcare team about your pain levels and any concerns you may have. They can adjust your pain management plan to ensure your comfort and safety during the recovery process. |
| 28 | How can I control nausea and vomiting after surgery? | Nausea and vomiting are common side effects after surgery, but there are several strategies that can help control these symptoms. Here are some tips for controlling nausea and vomiting after surgery:  1. Medications: Anti-nausea medications, such as ondansetron, metoclopramide, or dimenhydrinate, may be prescribed by your healthcare provider to help control nausea and vomiting.  2. Diet and hydration: Eating small, frequent meals and staying hydrated can help reduce the risk of nausea and vomiting. It's important to start with clear fluids and progress to solid foods gradually. 3. Relaxation techniques: Techniques such as deep breathing, meditation, and guided imagery can help reduce stress and promote relaxation, which can help reduce nausea and vomiting. 4. Acupressure: Acupressure, or the application of pressure to specific points on the body, may help reduce nausea and vomiting. This can be done by a trained practitioner or using acupressure bands.  5. Avoid triggers: Avoiding triggers, such as strong smells or certain foods, can help reduce the risk of nausea and vomiting.  6. Positioning: Positioning, such as keeping your head elevated or sitting upright, may help reduce nausea and vomiting. It's important to follow any instructions from your healthcare provider regarding nausea and vomiting after surgery. They can provide guidance on which medications and strategies are appropriate for you and how to manage any potential side effects or complications. | Nausea and vomiting are common side effects after surgery, often related to the anesthesia used during the procedure. There are several strategies to help control these symptoms:  1. Medication: Antiemetic medications can be prescribed to prevent or treat nausea and vomiting. Examples include ondansetron, metoclopramide, and dexamethasone. Inform your healthcare team if you are experiencing these symptoms, so they can provide appropriate medications.  2. Hydration: Sip small amounts of clear fluids, such as water, ice chips, or electrolyte replacement drinks, to help prevent dehydration caused by vomiting.  3. Gradual food intake: Start with clear liquids and slowly progress to solid foods as tolerated. Avoid heavy, greasy, or spicy foods initially. Stick to bland, easily digestible foods like crackers, toast, and applesauce.  4. Eating slowly: Take small bites and chew your food thoroughly. Eating slowly and in small portions can help reduce the likelihood of nausea and vomiting.  5 . Deep breathing and relaxation techniques: Practice slow, deep breaths and relaxation techniques, such as guided imagery or meditation, to help reduce anxiety and nausea. 6. Avoid lying down immediately after eating: Sit upright or elevate your upper body for at least 30 minutes to an hour after eating to help reduce the risk of nausea and vomiting. 7. Avoid strong smells: Strong odors can trigger nausea, so it's best to avoid them during your recovery.  8. Ginger: Ginger has natural antiemetic properties and can be consumed in various forms, such as ginger tea, ginger ale, or ginger chews, to help alleviate nausea. If your nausea and vomiting persist or worsen despite these interventions, contact your healthcare provider for further evaluation and guidance. They may recommend additional treatments or consider alternative causes of your symptoms. |
| 29 | How many hours after going up from the recovery room to the ward can I eat? | The amount of time you need to wait before eating after leaving the recovery room and being transferred to the ward can vary depending on several factors, such as the type of surgery, the type of anesthesia used, and how well you are recovering.  In general, you may be allowed to have clear liquids a few hours after the surgery, such as water, tea, or clear broth. Solid foods may be allowed after you have passed gas or have a bowel movement, which can take anywhere from 24 to 48 hours, or longer depending on your recovery.  However, it is important to follow your doctor's specific instructions regarding eating and drinking after surgery, as they may have specific recommendations based on your individual case. Your doctor may also restrict certain types of food or drink, especially if you have gastrointestinal issues or are at risk for complications.  So, it is best to ask your doctor or a member of the healthcare team for specific instructions about when you can eat after your surgery. They can provide you with personalized guidance and ensure that you are safely progressing in your recovery before resuming regular food and drink intake. | I am not a doctor, but generally, the time it takes for a patient to start eating after surgery varies depending on the type of surgery, the patient's condition, and their doctor's recommendations. After you've been transferred from the recovery room to the ward, your healthcare team will monitor your progress and provide guidance on when it is appropriate for you to start eating.  Typically, you may start with clear liquids, such as water, clear broth, or juice, before progressing to more solid foods. This process can take several hours or even a day or two, depending on your individual circumstances. It's essential to follow the advice of your healthcare team to ensure a safe and smooth recovery.  If you have concerns or questions about when you can eat after surgery, it is best to consult your surgeon or healthcare provider for personalized advice. |
| 30 | Sore throat after general anesthesia. What should I do? | A sore throat after general anesthesia is a common side effect and is usually caused by irritation to the throat from the breathing tube used during the procedure. Here are some strategies that can help alleviate a sore throat after general anesthesia:  1. Throat lozenges: Throat lozenges can help soothe a sore throat and reduce pain. Choose lozenges that contain a mild anesthetic, such as benzocaine, to numb the throat.  2. Saltwater gargle: Gargling with warm salt water can help reduce inflammation and soothe a sore throat. Mix a teaspoon of salt into a cup of warm water and gargle for 30 seconds before spitting it out.  3. Hydration: Staying hydrated can help alleviate a sore throat. Drink plenty of fluids, such as water, herbal tea, or clear broth, to keep the throat moist and reduce irritation.  4. Pain relievers: Over-the-counter pain relievers, such as acetaminophen or ibuprofen, can help alleviate pain and reduce inflammation. Follow the instructions on the label and do not exceed the recommended dose.  5. Rest: Resting the voice and avoiding speaking loudly or shouting can help reduce strain on the throat and promote healing.  6. Time: In most cases, a sore throat after general anesthesia will resolve on its own within a few days. If the sore throat persists or is accompanied by other symptoms, such as difficulty swallowing or breathing, contact your healthcare provider. It's important to follow any instructions from your healthcare provider regarding post-operative care and follow-up. They can provide guidance on how to manage a sore throat and any other potential side effects or complications after general anesthesia. | A sore throat after general anesthesia is a relatively common side effect, often caused by irritation from the endotracheal tube or laryngeal mask airway used during the procedure. To help alleviate discomfort and promote healing, you can try the following:  1. Stay hydrated: Drink plenty of water to keep your throat moist and help reduce irritation.  2. Gargle with warm saltwater: Dissolve half a teaspoon of salt in a glass of warm water, and gently gargle the solution for about 30 seconds before spitting it out. Repeat a few times a day to help soothe your throat.  3. Use over-the-counter pain relievers: Take over-the-counter pain relievers, such as acetaminophen or ibuprofen, as directed to help manage discomfort. Be sure to follow the recommended dosages and consult your doctor if you're unsure.  4. Suck on lozenges or hard candies: Sucking on throat lozenges or hard candies can help stimulate saliva production, which can help soothe your throat. Look for products with menthol or other soothing ingredients, but avoid giving them to young children due to the risk of choking.  5. Use a humidifier: Adding moisture to the air with a humidifier can help soothe a dry and irritated throat.  6. Get plenty of rest: Give your body time to recover from the surgery and anesthesia by getting adequate rest. If your sore throat worsens, persists for more than a few days, or is accompanied by difficulty breathing, high fever, or other concerning symptoms, it's important to contact your healthcare provider for further evaluation and guidance.  Note: This advice is for informational purposes only and should not replace a consultation with a medical professional. Always consult with your doctor or healthcare provider for personalized advice and recommendations. |

Questions corresponding to each category:

(1) General questions about anesthesia: 1, 2, 3, 4, 5, 7, 11, 26

(2) Preoperative preparation: 12, 13, 14, 15, 16, 17, 18, 24

(3) Pre-anesthetic evaluation: 8, 9, 10, 19, 20, 21, 22, 23, 25

(4) Postoperative recovery process: 6, 27, 28, 29, 30
